# Novel mutational landscapes and expression signatures of lung squamous cell carcinoma

**DOI:** 10.18632/oncotarget.23716

**Published:** 2017-12-27

**Authors:** Donghai Xiong, Jing Pan, Yuxin Yin, Hui Jiang, Eva Szabo, Ronald A. Lubet, Yian Wang, Ming You

**Affiliations:** ^1^ Cancer Center, Medical College of Wisconsin, Milwaukee, WI 53226, USA; ^2^ Department of Pharmacology and Toxicology, Medical College of Wisconsin, Milwaukee, WI 53226, USA; ^3^ Chemopreventive Agent Development Research Group, Division of Cancer Prevention, National Cancer Institute, Rockville, MD 20850, USA

**Keywords:** LUSC (lung squamous cell carcinoma), tumor evolution, somatic mutation, expression signature, immunotherapy

## Abstract

Lung squamous cell carcinoma (LUSC) is a major subtype of Non-Small Cell Lung Cancer. To increase our understanding of the LUSC pathobiology, we performed exome sequencing and RNA-seq in 16 murine carcinogen-induced LUSC tumors and 8 normal murine lung tissue samples. Additionally, we conducted single-cell RNA-seq on two independent tumors from the same murine model. We identified a list of 59 cancer genes recurrently mutated in the mice LUSC tumors, 47 (80%) of which were also mutated in human LUSCs. At the single cell level, we detected unique clonal mutation patterns for each of the two LUSC tumors, being initiated from clones carrying the mutant *Igfbp7* and *Trp53* genes, respectively. We also identified an expression signature serving as an effective classifier for LUSC tumors and a strong predictor of survival outcomes of lung cancer patients. Lastly, we found that some of the mutant LUSC genes were associated with the significantly altered tumoral expression of inhibitory immune checkpoint genes such as *PD-L1*, *VISTA*, *TIM3* and *LAG3* in human LUSCs. The novel findings of clonal evolution, mutational landscapes and expression signatures of LUSC suggested new targets for the overall LUSC therapy and the immunotherapy of LUSC.

## INTRODUCTION

Lung cancer is a major cause of cancer-related deaths worldwide, occurring in more than a million new patients annually [1]. Genetic factors, along with exposure to environmental carcinogens contribute significantly to the risk of developing lung cancer [2]. Non-Small Cell Lung Cancer (NSCLC) is the most common type of lung cancer, comprising 80–85% of all cases. Lung adenocarcinoma (LUAD) and lung squamous cell carcinoma (LUSC) are the two major subtypes of NSCLC, each comprising about 30% of lung cancer diagnosis. The tumor genomics profiles between LUAD and LUSC differed significantly and high heterogeneity was observed within each cancer type [3–5]. Therapies for LUAD are often ineffective for LUSC [6]. LUSC is a highly heterogeneous disease that develops via multiple complex steps [7, 8]. Genetic etiology of LUSC has been studied extensively [9–11], however, more research is needed to increase the knowledge base of LUSC and design more effective prevention and therapeutic strategies.

A mouse model of LUSC in which the tumors are induced by the carcinogen, *N*-nitroso-tris-chloroethylurea (NTCU), was developed and widely used in chemoprevention studies of LUSC [12]. However, the underlying genetic profiles of this murine model and its resemblance to human LUSCs have not been well characterized. Because carcinogen-induced mouse models have become important for studies of oncoimmunology [13], it is necessary to systematically analyze the mutation and transcriptome profiles of the NTCU-induced murine LUSC model and compare them to the corresponding profiles of human LUSCs [14, 15] to identify the genes with potential clinical value for further study. In the present study, we assessed the mutational and transcriptional characteristics of mice LUSCs. We identified the the novel patterns of clonal evolution, mutational landscapes and expression signatures of LUSC, which could contribute to the development of new LUSC therapeutic strategies.

## RESULTS

### Whole-exome sequencing (WES) of mouse lung squamous cell carcinoma (LUSC) tumors

To characterize the genetic alterations that occur in mouse LUSCs, we performed WES in 16 surgically resected NTCU-induced NIH Swiss mouse LUSC tumors, along with 8 normal mouse lung tissues. LUSC tumors and normal lung tissues were sequenced to an average read depth of 129 X (range: 98 X to 165 X) and 137 X (range: 112 X to 179 X), respectively (Supplementary Table 1). The 16 mouse LUSCs harbored 5,664 somatic coding mutations (mean = 354) that consisted of 2,885 missense, 106 nonsense, 2,426 silent mutations, 167 small insertions and deletions (indels) and 80 alterations residing in exon-exon boundaries (Supplementary Table 2 [Excel File]). The most common substitutions were C>T transversions (Supplementary Figure 1), indicative of oxidative DNA damage [16].

### WES identified recurrently mutated cancer genes and clonal mutations in mouse LUSC

**Figure 1:**
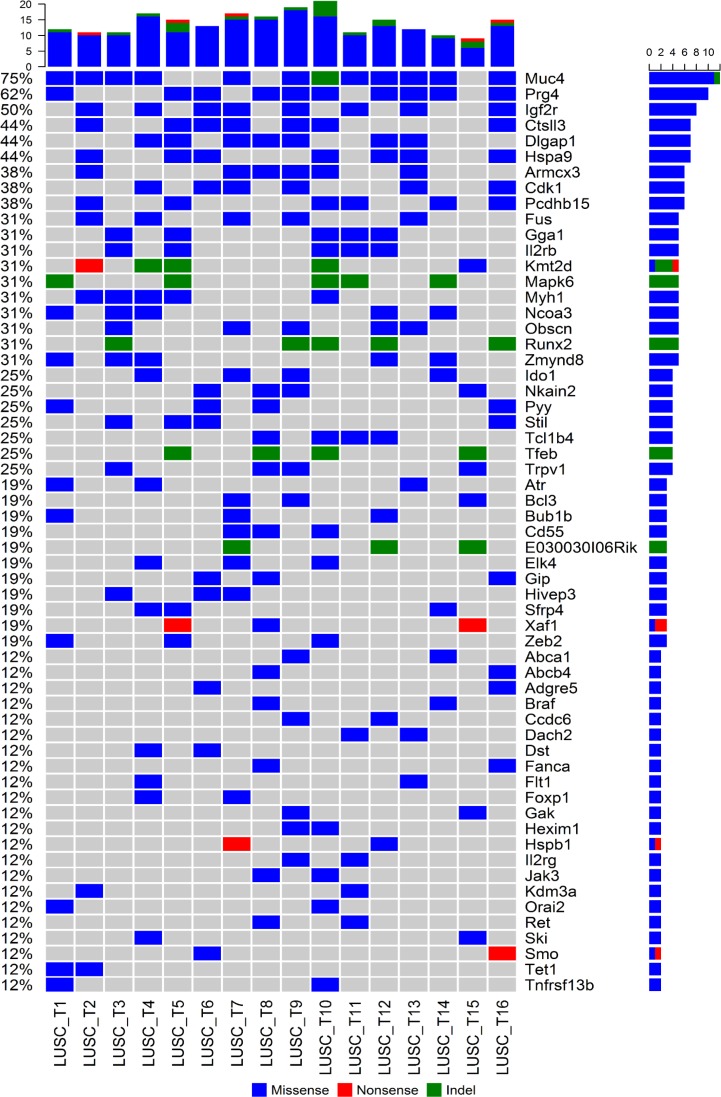
Profiles of recurrently mutated cancer genes in mouse LUSC tumors The non-silent somatic mutation spectrum of the 59 cancer genes that were recurrently mutated in the 16 mice LUSC tumors was plotted. The order of genes was sorted by the mutation frequency decreasingly. The top bar plot showed the total number of mutations in any of the 59 genes per sample, the right-side and the left-side legends showed the number and frequency of mutations within each gene across the 16 mouse LUSC tumors.

Identification of cancer genes that are recurrently mutated in mouse LUSC tumors (mutated in at least two tumors) may reveal the novel genetic mechanisms of carcinogenesis and progression of LUSC. Therefore, we analyzed the mutation profiles of the mouse LUSCs focusing on the comprehensive list of 2,027 cancer related genes (from the file “allOnco_Feb2017.tsv” available at http://www.bushmanlab.org/links/genelists). It was found that 59 cancer genes were recurrently mutated in our mouse LUSC tumors (Figure 1). The cancer genes most frequently mutated in the mouse LUSC tumors (≥25%, i.e., mutated in at least 4 samples of 16 tumors) were as follows: *Muc4, Prg4, Igf2r, Ctsll3, Dlgap1, Hspa9, Armcx3, Cdk1, Pcdhb15, Fus, Gga1, Il2rb, Kmt2d (Mll2), Mapk6, Myh1, Ncoa3 (Src3), Obscn, Runx2, Zmynd8, Ido1, Nkain2, Pyy, Stil, Tcl1b4, Tfeb,* and *Trpv1* (Figure 1).

**Figure 2:**
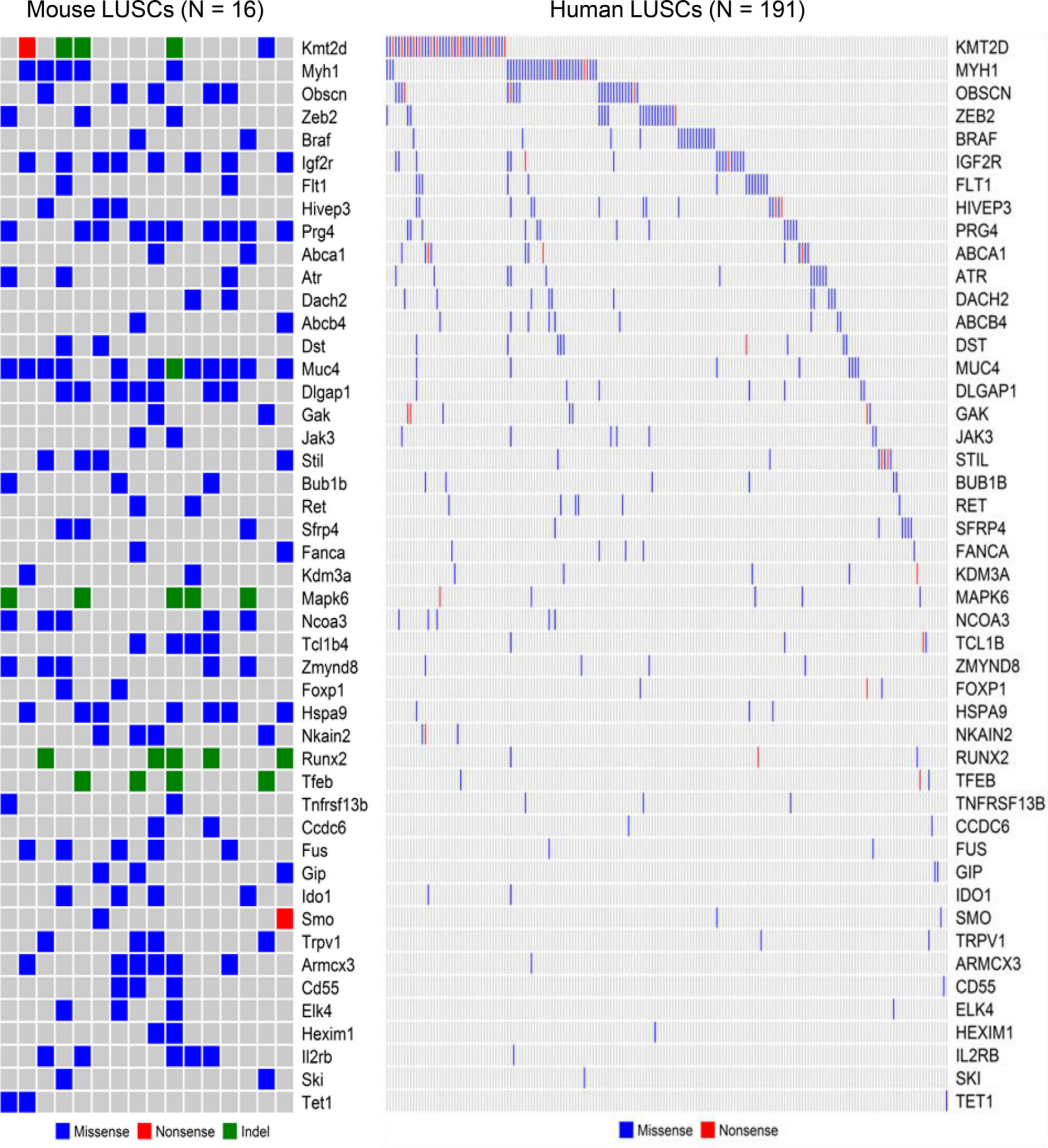
Comparison study of mouse and human LUSC genes In terms of non-silent somatic mutations, 47 of the 59 cancer genes recurrently mutated in the mouse LUSCs were also mutated in the human LUSCs archived in the COSMIC database. The waterfall plots showed the side-by-side comparison of the 47 mutated LUSC genes shared between mouse and human.

To identify whether the 59 recurrently mutated cancer genes in mouse LUSC tumors were also mutated in human LUSC tumors, we downloaded the somatic mutation profiles of 191 human LUSC tumors that are accessible through COSMIC database (http://cancer.sanger.ac.uk/cosmic) and performed cross-species comparisons. We found that 47 of the 59 cancer genes recurrently mutated in the mouse LUSCs were also mutated in the human LUSCs (Figure 2).The mutation profiles of most of these 47 genes were mutually exclusive across the human LUSC tumors and they comprehensively characterized the human LUSC tumors (Figure 2). Among the 47 commonly mutated cancer genes shared between mouse and human LUSCs, the frequently mutated ones in human LUSCs include *KMT2D* (*MLL2*), *MYH1*, *OBSCN*, *ZEB2*, *BRAF*, *IGF2R*, *FLT1*, *HIVEP3*, *PRG4*, *ABCA1*, *ATR*, *DACH2*, *ABCB4*, *DST*, and *MUC4* (Figure 2). The proportion of the cancer genes recurrently mutated in mice LUSCs that were also mutated in human LUSCs was as high as 80% (= 47/59), suggesting that the NTCU-induced mouse LUSC model is an excellent model to study the genetic mechanisms of human LUSC. However, about 20% (= 12/59) of recurrently mutated cancer genes of mouse LUSCs were not found to be mutated in human LUSCs, suggesting either the unique mechanisms of LUSC carcinogenesis in mice or the necessity to sequence more human LUSC tumors to enlarge the mutation spectrum of human LUSCs.

In addition, our analysis revealed that each LUSC tumor had a sample-specific mutational landscape consisting of a mixture of recurrent and private clonal mutations (Supplementary Figure 2). The clonal mutation spectra across the 16 mouse LUSCs were shown in Supplementary Figure 3 and Supplementary Table 3. There were nine most frequent clonal mutations identified in at least 4 of 16 LUSC tumors, including *Hspa9*: A651S (7), *Cdk1*: S39I (6), *Pcdhb15*: R461C (6), *Ctsll3*: P329S (5), *Gga1*: D358N (5), *Il2rb*: R475S (5), *Dlgap1*: A329T (4), *Nkain2*: V67L (4), and *Pyy*: P42L (4).

### scRNA-seq (single-cell RNA-sequencing) identified clonal mutations in the two mice LUSC tumors

**Figure 3:**
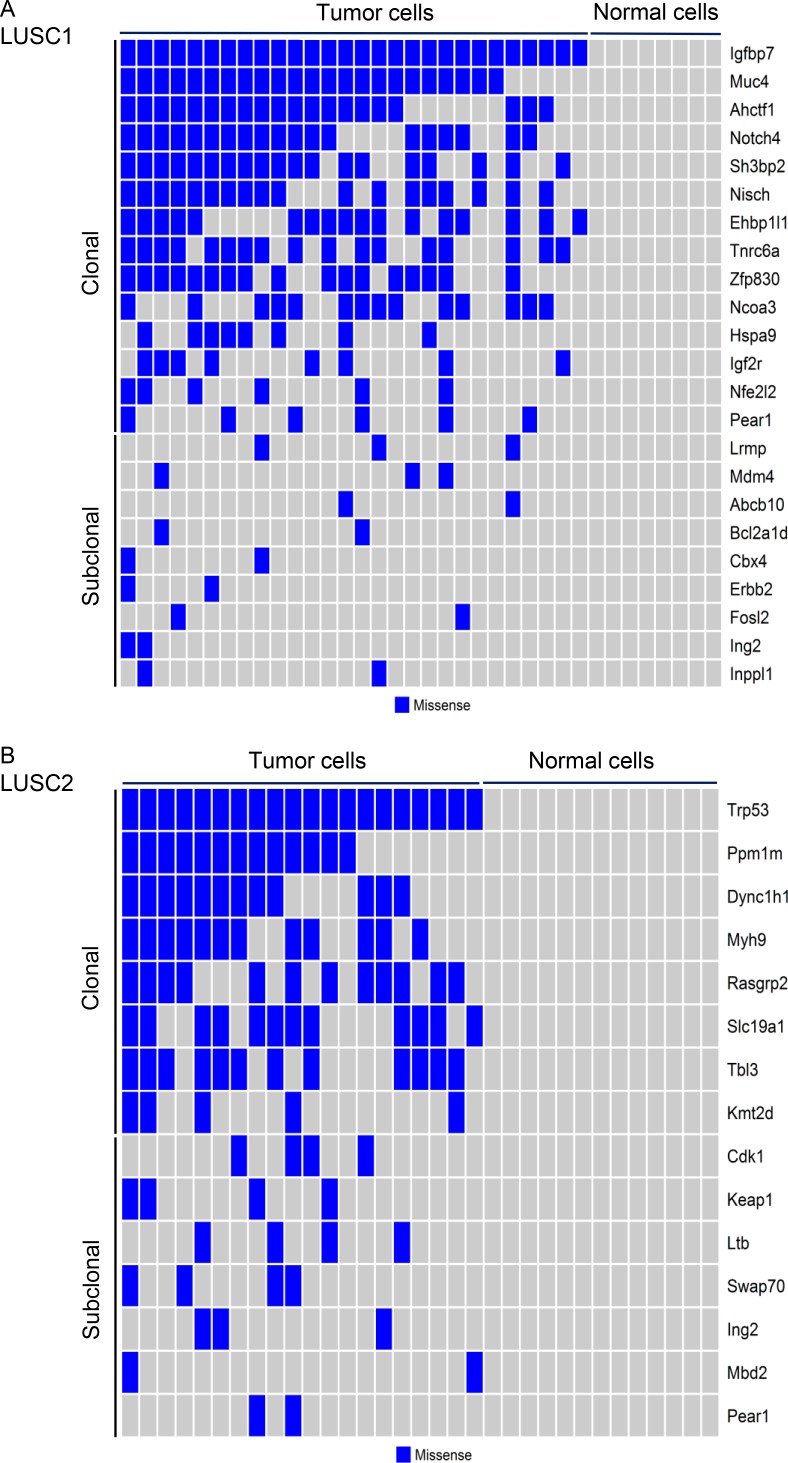
Clonal and subclonal mutations across the tumor cells from the two mouse LUSC tumors The somatic missense mutations in the cancer related genes of single tumor cells from LUSC1 (**A**) and LUSC2 (**B**) were plotted. The corresponding normal cells did not carry any mutations.

Single-cell analysis by scRNA-seq was used to characterize the nonsilent somatic mutations in two mouse LUSC tumors, with a specific focus on the known cancer genes or the mutated genes identified by our exome-seq of mouse LUSC tumors. Single tumor and normal cells were differentiated from one another based on whether any mutations in the above genes can be identified. With respect to the first mouse LUSC tumor (LUSC1), 36 single cells from LUSC1 were classified into 28 tumor cells and 8 normal cells according to the mutation status of the cancer genes (Figure 3A). The 33 single cells from the second mouse LUSC tumor (LUSC2) were classified into 20 tumor cells and 13 normal cells according to the mutation status of the cancer genes (Figure 3B). The details of the cancer gene mutations of the two mouse LUSC tumors can be seen in Supplementary Table 4.

Next, we inferred the clonal evolutionary history of the two mouse LUSC tumors (Supplementary Figures 4 and 5). For LUSC1, all the single tumor cells had the clonal mutation (R45P) in *Igfbp7* and a subset of single tumor cells had the mutation (R2457S) in *Igf2r* (Figure 3A). IGFBPs participate in a complex signaling axis called IGF-IGFR-IGFBP. In addition, the genes having somatic mutations in LUSC1 involve a number of transcription regulators such as *Ahctf1*, *Notch4*, *Ncoa3*, *Nfe2l2* (Figure 3A). The scRNA-seq of LUSC2 identified a *Trp53* somatic missense mutation (Q97L) in all the tumor cells (Figure 3B). Somatic mutations of the LUSC2 tumor were also found in a number of novel driver genes such as *Myh9*, *Kmt2d* and *Keap1* (Figure 3B).

### The activation of a common cancer gene expression module in the two mouse LUSC tumors

**Figure 4:**
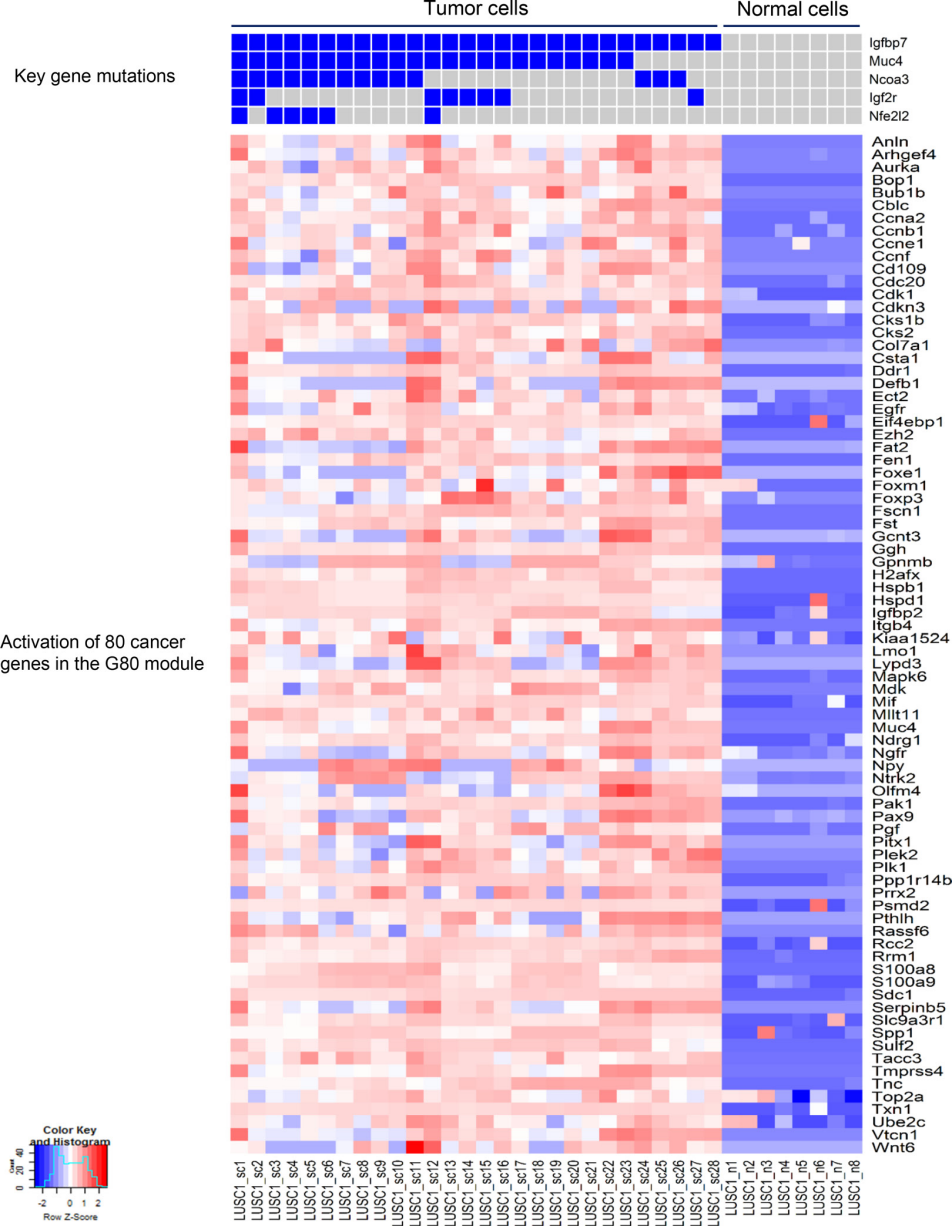
Mutation profiles of the key cancer genes and the corresponding up-regulated cancer genes in LUSC1 single tumor cells The nonsilent mutations in the key genes correlated with significant upregulation of the expression of 80 cancer genes in the G80 module in the single tumor cells from the mouse LUSC1 tumor.

**Figure 5:**
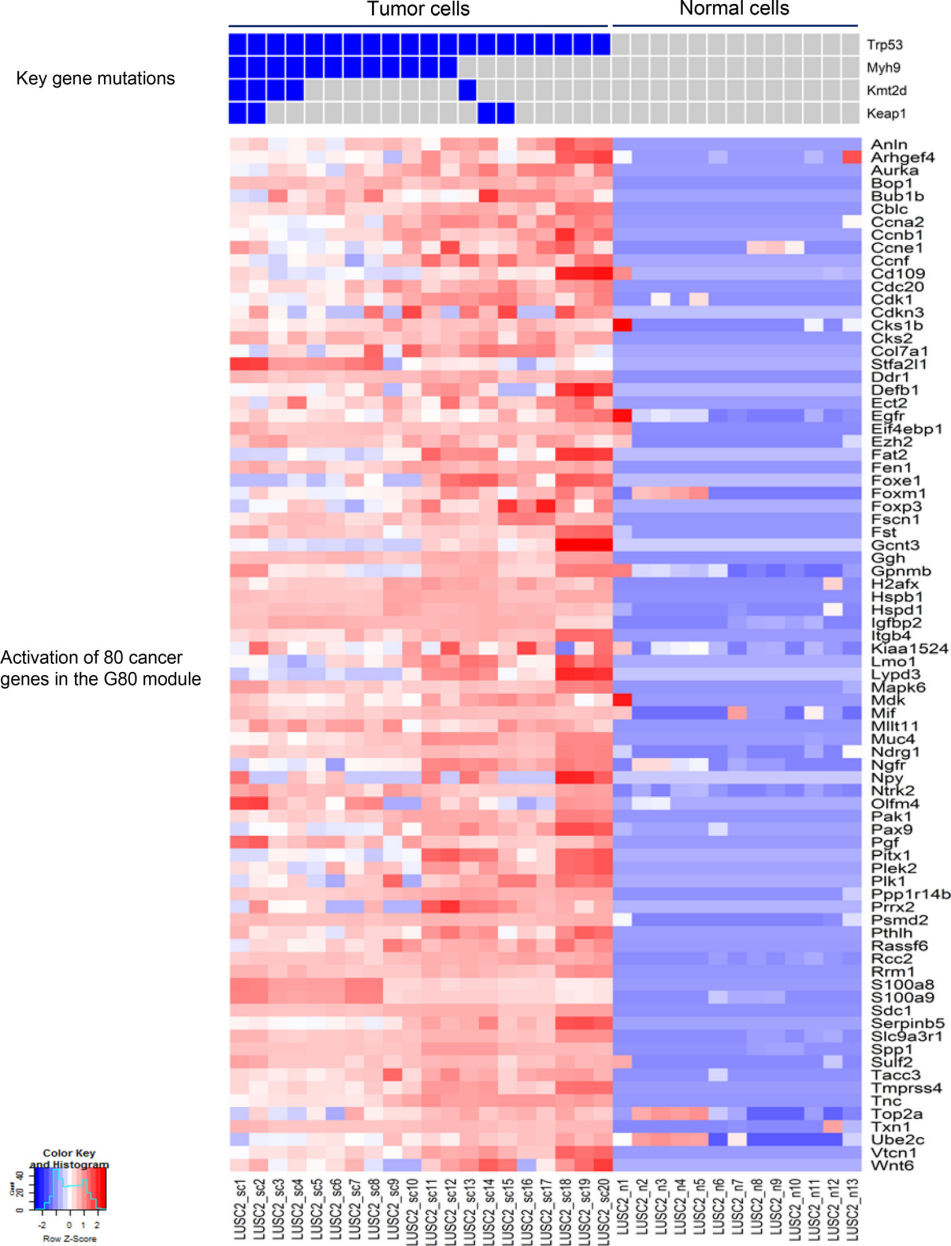
Mutation profiles of the key cancer genes and the corresponding up-regulated cancer genes in LUSC2 single tumor cells The nonsilent mutations in the key genes correlated with the significant upregulation of the expression of 80 cancer genes in the G80 module in the single tumor cells from the mouse LUSC2 tumor.

**Figure 6:**
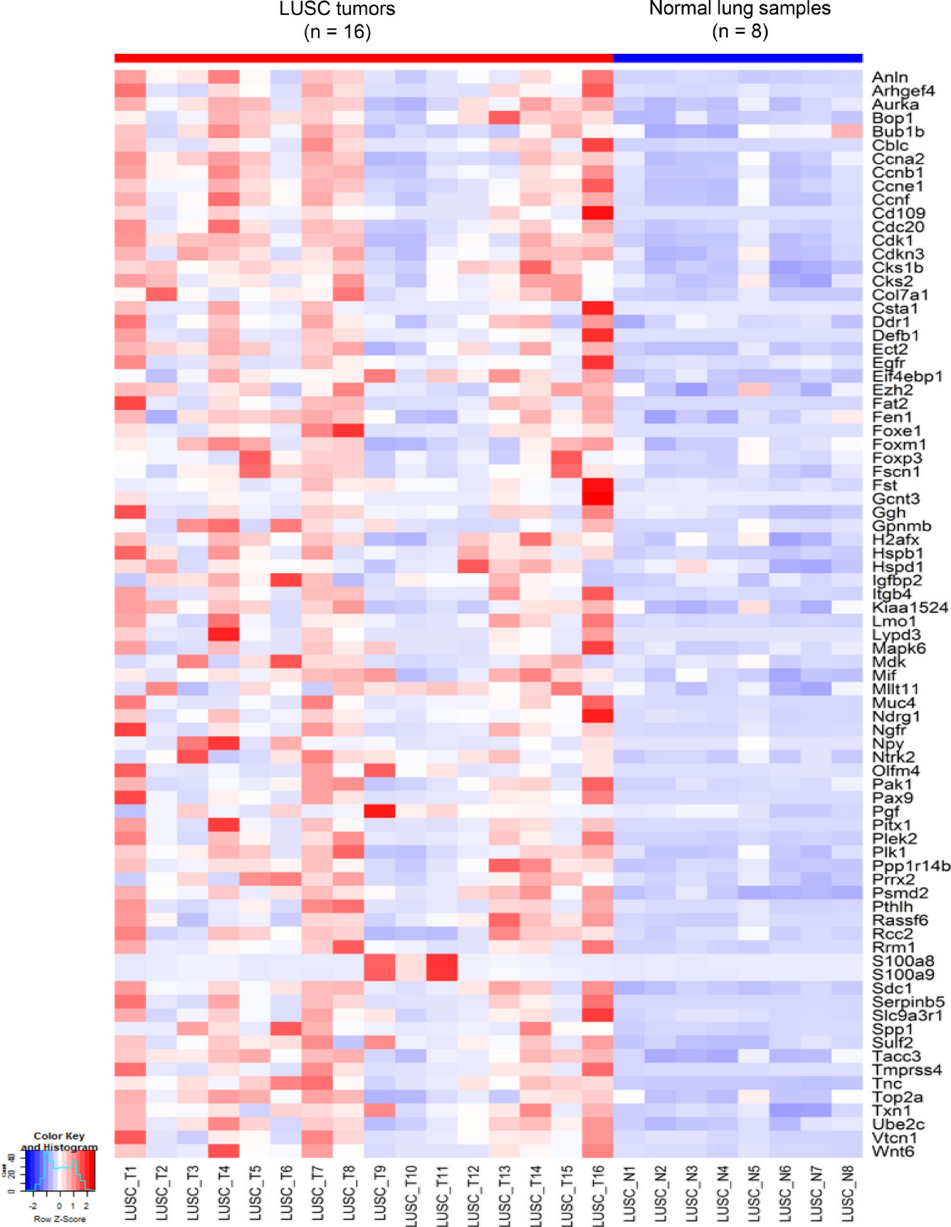
Heatmap of G80 expression in the 16 mouse LUSC tumors versus 8 control normal lung samples At the bulk inter-tumoral level, the expression patterns of the 80 cancer genes in the G80 module still clearly separated the mouse LUSC tumors from the control normal lung tissues.

**Figure 7:**
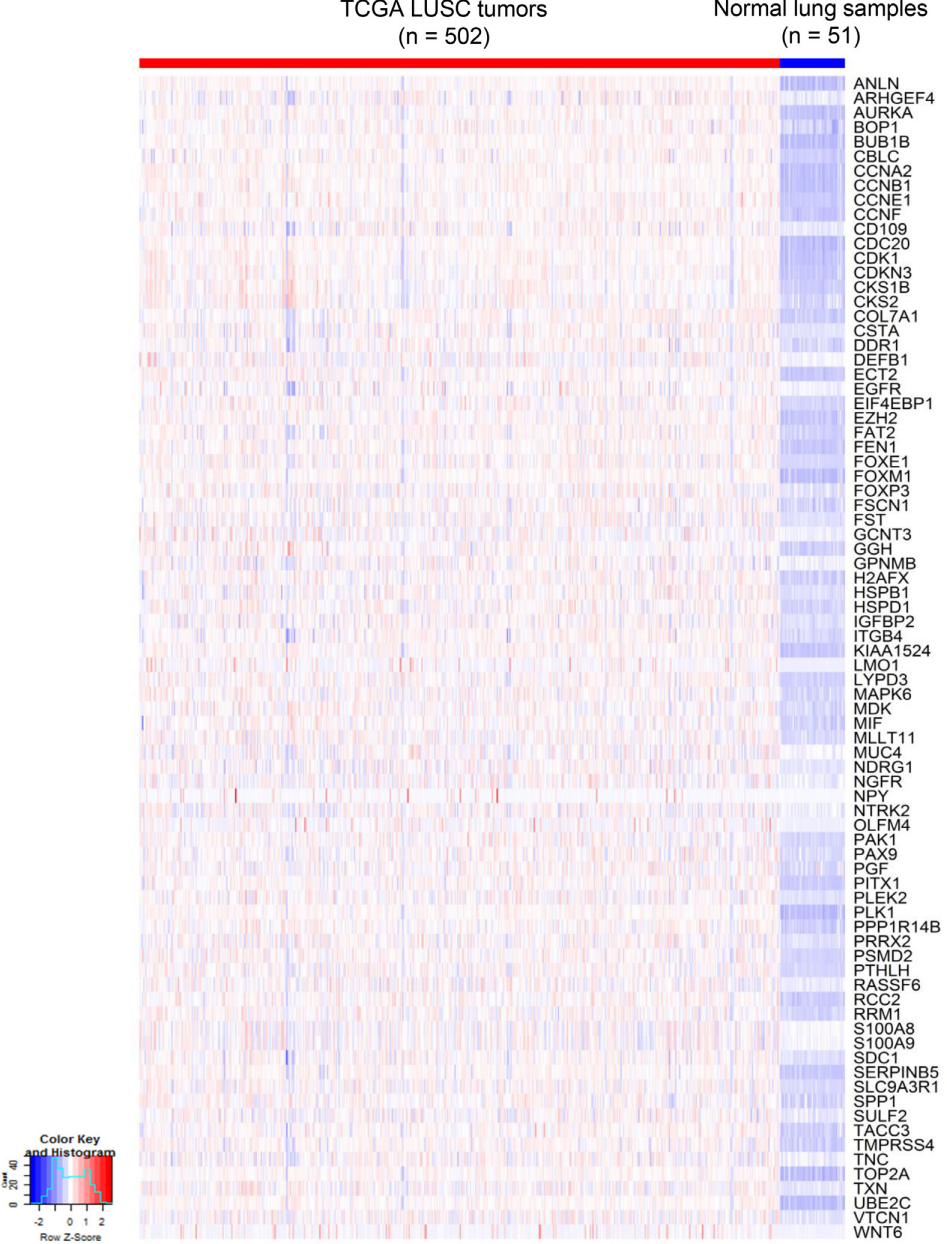
Heatmap of G80 expression in 502 TCGA LUSC tumors versus 51 control normal lung samples At the bulk inter-tumoral level, the G80 gene expression signature that distinguished between single cells in mouse LUSC model also clearly differentiated the same sub-groups for the TCGA LUSC samples.

Based on the mutation spectra of the two LUSC tumors, differential expression (DE) analysis was performed between tumor and normal cells in each tumor. Interestingly, DE analysis and comparison study of the two LUSC tumors identified a common set of 80 cancer genes termed ‘G80’ that were activated in both the LUSC1 and LUSC2 tumor cells although they had a different set of key cancer gene mutations (Figures 4 and 5). The G80 geneset was further tested in the mouse and human LUSC tumors at the bulk sample level. Analysis of the 16 mice LUSC tumors and 8 control normal lungs showed that the G80 module expression could differentiate the tumor samples from the normal lung samples (Figure 6). We also analyzed the G80 module cancer gene expression in the 502 human LUSCs and 51 control normal lung samples from TCGA. Interestingly, the G80 gene expression signature clearly differentiated LUSC tumors from normal samples again (Figure 7). These data suggested that the G80 module can serve as a classifier for LUSC tumors in both mouse and human samples.

**Figure 8:**
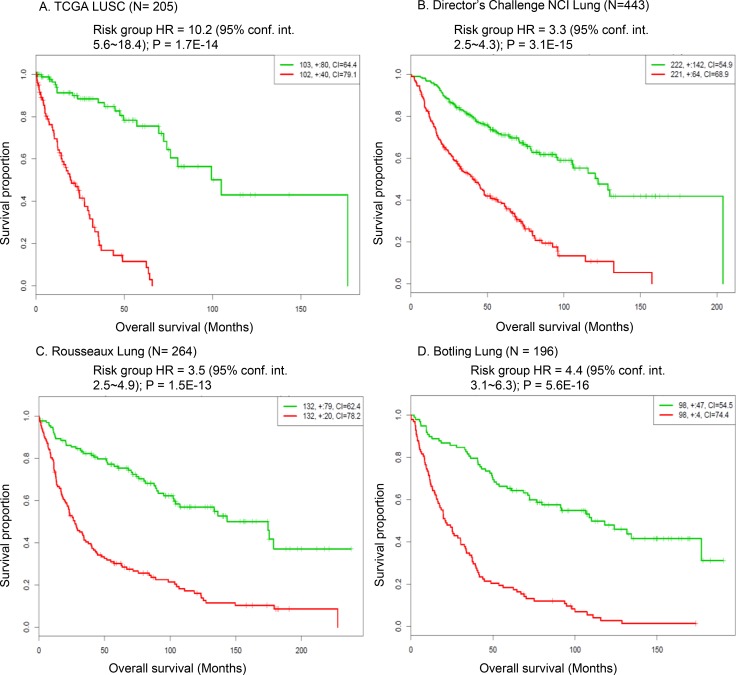
Survival analysis of the G80 module in human LUSC and NSCLC patient cohorts Kaplan-Meier analysis showing that the expression signature of the G80 cancer gene module was significantly associated with the overall survival outcome in the TCGA LUSC cohort and the other three NSCLC patient cohorts. Red curve denoted high risk group based on the G80 module expression, green curve denoted low risk group based on the G80 module expression.

Next, we sought to explore whether the expression signature of the G80 cancer gene module was associated with survival outcome in lung cancer patients. The TCGA LUSC cohort and the other three independent human NSCLC gene expression data sets were analyzed [17–20]. The high-risk gene expression signature of the G80 cancer gene module was significantly associated with poor overall survival outcome across these four large and representative human lung cancer datasets (Figure 8). The risk group hazard ratio (HR) based on G80 module expression in the TCGA LUSC cohort was 10.2 (*P* = 1.7E–14), which was much greater than the HR values of the other three NSCLC cohorts (HR = 3.3, 3.5, 4.4, Figure 8). This reflected that the G80 module was developed from LUSC samples so its performance was better in the LUSC cohort than in the NSCLC cohort which also contains LUAD (lung adenocarcinoma) samples. Our data suggested that the overall expression of the G80 module genes can be used as a biomarker to assess the survival outcomes of lung cancer patients, especially patients with LUSC.

### Associations of mutant LUSC genes with the immune checkpoint gene expression in human LUSCs

**Figure 9:**
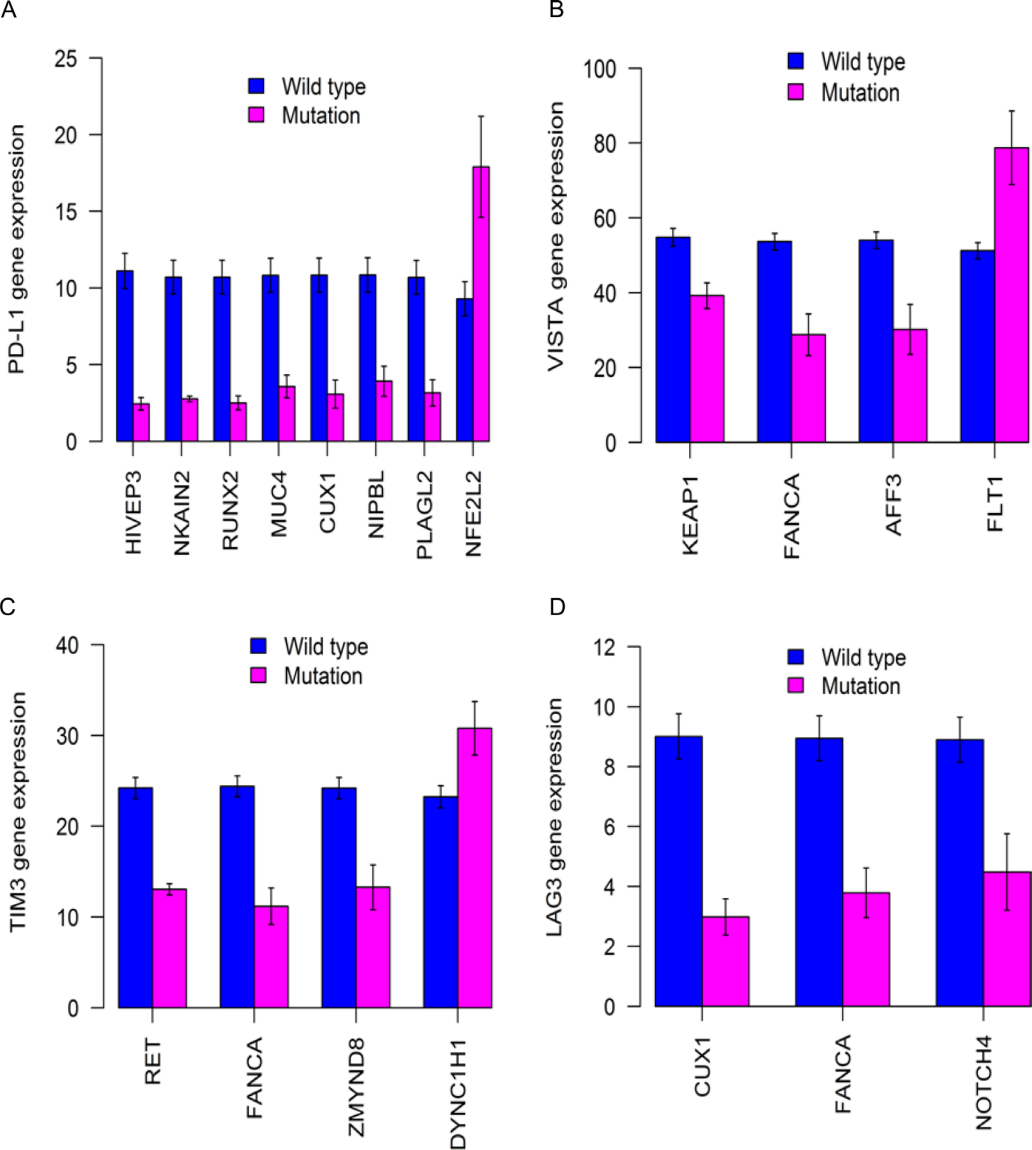
Differential expression of immune markers in 176 TCGA LUSCs with significantly mutated cancer genes Different mutant genes identified from sequencing of mouse LUSC tumors were associated with significantly decreased expression of tumoral PD-L1 (**A**), VISTA (**B**), TIM3 (**C**), and LAG3 (**D**). All the *P* values < 0.05 after multiple testing adjustment.

Our comprehensive analysis yielded a list of cancer genes with recurrent or clonal mutations for LUSC (Figures 1–3, Supplementary Figure 3, and totaling 106 genes). In order to test whether these mutated genes have potential clinical application, the associations between mutational status of the identified LUSC genes and gene expression of immune checkpoint genes were analyzed by using a set of 176 TCGA LUSC tumor samples. We examined the expression of inhibitory immune checkpoint genes, including *CTLA4*, *PD-1* (*PDCD1*), *LAG3*, *TIM3* (*HAVCR2*), *PD-L1* (*CD274*), and *VISTA* (*C10orf54*). The suppressed tumoral *PD-L1* expression was significantly associated with mutations in eight genes (Figure 9, Supplementary Table 5): *HIVEP3* (*P* = 2.8E-11), *NKAIN2* (*P* = 2.9E-11), *RUNX2* (*P* = 2.5E-09), *MUC4*(*P* = 2.3E-06), *CUX1* (*P* = 9.0E-06), *NIPBL* (*P* = 6.1E-05), *PLAGL2* (*P* = 0.0001), and *NFE2L2* (*P* = 0.019). The mutant *NFE2L2* was associated with increased tumoral *PD-L1* expression while the other seven mutant genes associated with decreased *PD-L1* expression. The tumoral *VISTA* expression was significantly decreased in the LUSCs with mutations in the *KEAP1* (*P* = 0.0005), *FANCA* (*P* = 0.0076), and *AFF3* (*P* = 0.008) genes while increased in the LUSCs with the mutant *FLT1* gene (*P* = 0.02). Moreover, tumoral *TIM3* was significantly decreased in the mutant *RET* (*P* = 4.2E-10), *FANCA* (*P* = 0.0007), or *ZMYND8* (*P* = 0.028) LUSC tumors while increased in the mutant *DYNC1H1* (*P* = 0.027) LUSC tumors. The suppressed tumoral *LAG3* expression was significantly associated with mutations in *CUX1* (*P* = 6.9E-07), *FANCA* (*P* = 0.0005) or *NOTCH4* (*P* = 0.028) genes.

**Figure 10:**
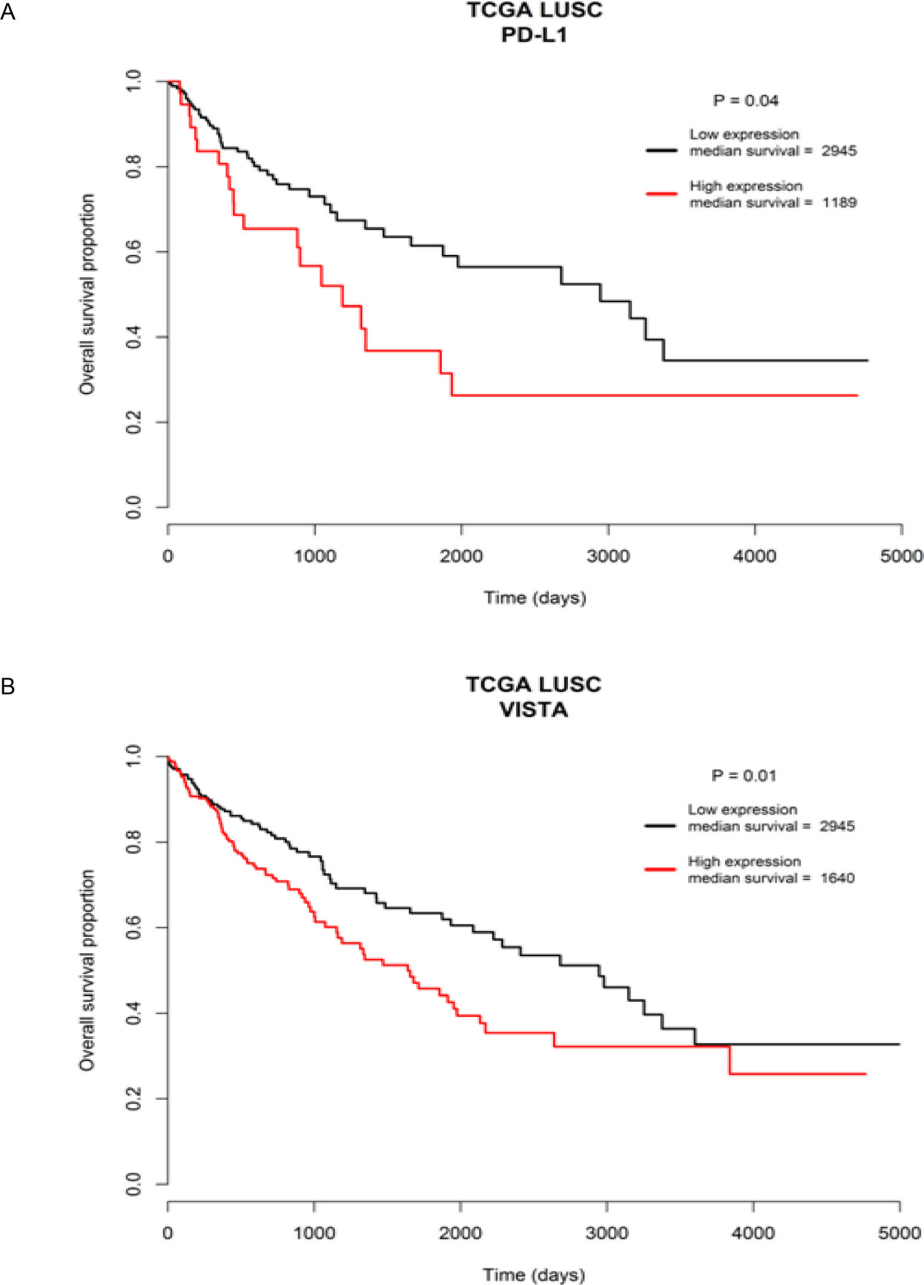
Survival outcome analysis based on immune checkpoint gene expression Worse overall survival outcome was significantly associated with overexpressed tumoral *PD-L1* gene expression group (**A**), and overexpressed tumoral *VISTA* gene expression group (**B**), relative to the down-regulated expression group of the two genes, respectively.

In addition, the LUSC patients with overexpressed *PD-L1* or *VISTA* had significantly worse overall survival outcome compared to the patients with decreased expression of *PD-L1* or *VISTA* (Figure 10). The median overall survival time of high *PD-L1* vs. low *PD-L1*expression group was 1189 days vs. 2945 days (*P* = 0.04) and of high *VISTA* vs. low *VISTA* expression group was 1640 days vs. 2945 days (*P* = 0.01) (Figure 10). These data suggested that aberrant immune checkpoint gene expression could have significant impact on the survival outcome of LUSC patients.

## DISCUSSION

We identified 59 cancer genes that are recurrently mutated in mice LUSC tumors (Figure 1), 47 of which (80%) were also mutated in the human LUSCs (Figure 2). Most of the commonly mutated genes in both mouse and human LUSCs have genetic and/or functional significance to lung cancer, such as *Kmt2d (Mll2)*, *Zeb2*, *Braf*, *Igf2r, Flt1, Atr, Muc4, Ncoa3 (Src3),* and *Ido1*. KMT2D, the key enzyme governing histone modification, is a well-known driver gene with a high somatic mutation rate in LUSC tumors [14, 21]. The mutant *KMT2D* significantly associated with poor prognosis of LUSC [14]. ZEB2 expression increased in NSCLC [22] and it can induce epithelial-mesenchymal transition (EMT) to facilitate the metastasis of cancer cells [23, 24]. BRAF mutated lung cancer is a genetically distinct subtype that accounts for about 5% of NSCLC [25]. *IGF2R* is mutated frequently in LUSC and it suppresses cancer cell growth [26]. NSCLC patients with low IGF2R expression had a poorer prognosis than those with high IGF2R expression [27]. IGF2R inhibition in NSCLC cell lines resulted in increased proliferation, migration and invasion abilities and a reduced apoptosis rate of the cancer cells [27]. The functional FLT1 variant and FLT1 mRNA expression are prognostic determinants of patient survival and recurrence in stage I-III NSCLC [28, 29]. *ATR* encoded protein kinase is a master regulator of the DNA-damage response [30] and its genetic alteration was associated with lung cancer risk [31]. MUC4 has been shown to play a tumor-suppressor role in NSCLC by altering p53 expression [32]. A reduced expression of *MUC4* was observed in LUSC tumors, especially in advanced tumor stages [32, 33]. A high somatic mutation rate in *MUC4* was found in smokers having NSCLC, suggesting that the *MUC4* gene is a potential target of nicotine in causing NSCLC [34]. This is supported by our data revealing *Muc4* as one of the most recurrently mutated genes in mice LUSC. *NCOA3* may be an oncogene for lung cancer due to its role in promoting lung cancer cell invasion [35, 36]. *IDO1* is an immune checkpoint gene involved in lung cancer progression and metastasis [37, 38] and thus a promising target for lung cancer immunotherapy.

Furthermore, we detected clonal mutations in the 16 mice LUSC tumors. Each tumor had a unique set of clonal mutations (Supplementary Figures 2 and 3), supporting previous findings of distinct clonal mutation structures across the same type of NSCLC tumors [39]. Although the overall clonal mutation spectra were different across the 16 mice LUSC tumors, a number of recurrent clonal mutations were identified (Supplementary Figure 3, Supplementary Table 3). The most frequent clonal mutations were found in the following cancer genes: *Hspa9*, *Cdk1*, *Pcdhb15*, *Ctsll3*, *Gga1*, *Igf2r*, *Il2rb*, *Dlgap1*, *Nkain2*, and *Pyy*. HSPA9 (mortalin/GRP75/PBP74), was overexpressed in different tumor types and detected in different subcellular compartments of cancer cells, indicating its functional role in cancer [40, 41]. *HSPA9* may play an important role in the progression of lung carcinoma by regulating the expressions of p53 and bcl-2 [42]. CDK1 is essential for cell cycle and its overexpression is directly correlated with the clinical features such as tumor stage and therapy outcome [43]. CTSL (*Ctsll3* in mice*)*, a lysosomal acid cysteine protease, is known to play important roles in tumor metastasis and resistance to chemotherapy [44]. *IL2RB* gene variants have been associated with human lung cancer risk in a large patient population [45]. *NKAIN2* is a novel tumor suppressor gene [46–49]. The recurrent somatic clonal mutations of these genes in LUSC tumors suggest that they could be the therapeutic targets of LUSC treatment.

Tumor progression is a dynamic evolutionary process acting at the level of individual cells. A tumor typically arises from a single progenitor cell whose founder mutation gives it a growth advantage over the surrounding cells and helps it to evade the immune response. Consequently, the clone arising from this cell expands and, over time, the descendant cells develop further into subclones by acquiring additional somatic mutations [50]. The mouse LUSC1 tumor was initiated from clonal cells having a mutant *Igfbp7* gene, which may lead to an aberrant Igf-Igfr-Igfbp axis that is an important driver of cancer [51]. *Igfbp7* is a tumor suppressor gene inactivated in lung cancer by DNA hypermethylation and it is regulated by p53 [52]. Aberrant methylation and downregulation of *IGFBP7* were frequently observed in NSCLCs [53, 54]. Our findings provided evidence indicating that non-silent somatic mutations in *IGFBP7* could be the important driver alteration leading to LUSCs. Another important driver gene for LUSC1 is *Nfe2l2* in the Keap1-Nrf2 pathway. Mutations in *NFE2L2* could cause the excessive intracellular accumulation of NFE2L2 protein and the subsequent activation of downstream oncogenes resulting in tumor growth promotion [55].

For LUSC2, the founder clone was driven by mutant *Trp53*, the mouse homolog of the human *TP53* gene that is a well known driver gene frequently mutated in the human LUSCs [14, 56]. In addition, a number of driver genes, such as *Myh9*, *Kmt2d* and *Keap1*, accumulated mutations in the subclones of LUSC2. The mutations or abnormal expression of these genes have been linked to aberrant molecular events leading to cancer, such as the interruption of p53 stabilization (*MYH9*) [57], abnormal chromatin/histone modification (*KMT2D*) [58], and aberrant Keap1-Nrf2 pathway (*KEAP1*) activity [55, 59]. *Myh9*, which encodes nonmuscle myosin IIa, has been identified as tumor suppressor of squamous cell carcinomas (SCCs) [57]. *Myh9* knockout triggers invasive formation of SCCs. Myh9 plays a role in regulating posttranscriptional p53 stabilization and it is frequently mutated and diminished in human SCCs [57]. Kmt2d serves as the key enzyme in histone lysine methyltransfer and thus is one of the important epigenetic regulators whose mutations could lead to the development of tumors with abnormal histone modifications [58]. Human studies revealed a high frequency of non-silent somatic mutations within *KMT2D* for LUSC tumors [14, 21]. Moreover, mutant *KMT2D* was the alteration most significantly associated with poor prognosis of LUSC [14]. In human cancers, similar to *NFE2L2*, *KEAP1* mutations would disrupt the normal combination of NFE2L2 and KEAP1 in tumors, resulting in accumulation of excessive intracellular NFE2L2 and activation of its downstream genes and the eventual promotion of tumor growth [55]. The Keap1-Nrf2 pathway is important for cytoprotection from oxidative stress. The discrepancy from normal in the Keap1-Nrf2 pathway may lead to promotion of tumor [59].

In addition, scRNA-seq identified a set of cancer genes; i.e., the G80 module that is an effective classifier for LUSC tumors in both mice and humans and an effective biomarker to assess the survival outcomes of lung cancer patients. Pathway enrichment analysis of the G80 module cancer genes showed that these genes are significantly enriched in six cancer related molecular pathways, including Cell cycle, PI3K-Akt, p53 and ErbB signaling, Focal adhesion and ECM-receptor interaction pathways (Supplementary Figure 6, Supplementary Table 6). Alteration of transcripts in these pathways may be a common mechanism involved in LUSC tumorigenesis and progression. The G80 module was activated in both LUSC1 and LUSC2 tumor cells although the two tumors had different clonal mutations. This is not surprising because the major driver genes in the two tumors functionally interacted with each other and played similar roles in the same pathway. For example, IGFBP7 might be regulated by TP53 in lung cancer cells [52] and mutant *NFE2L2* or *KEAP1* led to similar functional consequences in terms of causing NFE2L2 accumulation and aberrant Keap1-Nrf2 pathway activity in cancer cells [59].

Lastly, we found that the mutant LUSC genes could be associated with the significantly altered tumoral expression of inhibitory immune checkpoint genes such as *PD-L1*, *VISTA*, *TIM3* and *LAG3*. These findings suggest that immune checkpoint signaling activity could be significantly altered by mutations in LUSC genes identified in this study. We observed both up- and down-regulated immune checkpoint gene expression patterns according to different mutant genes in LUSC tumors (Figure 9). Notably, suppressed tumoral expression of immune checkpoint genes has been associated with mutated oncogenes in lung cancer [14, 60]. Particularly, the increased tumoral *PD-L1* gene expression was significantly associated with the mutant *NFE2L2* gene (Figure 9). This supported the finding that PD-L1 expression in LUSC tumor cells was associated with *NFE2L2* mutations [61] and indicated that LUSC patients carrying *NFE2L2* mutations may be more responsive to anti-PD-L1 immunotherapy. Similarly, the mutant *FLT1* and *DYNC1H1* genes associated with higher tumoral gene expression of *VISTA* and *TIM3*, respectively, suggesting that they may serve as biomarkers to predict the effectiveness of VISTA-targeted and TIM3-targeted antitumor immunotherapy for LUSC patients. Moreover, we detected that the tumoral overexpression of *PD-L1* and *VISTA* were significantly associated with worse survival outcome (Figure 10). This suggested that the mutant genes such as *NFE2L2* and *FLT1* associating with higher expression of *PD-L1* and *VISTA* may be correlated with worse survival outcome. Interestingly, we found the trends of the associations with worse overall survival outcome for the *NFE2L2* and *FLT1* mutations (Supplementary Figure 7). The association of *NFE2L2*, *FLT1* and *DYNC1H1* mutations with higher expression of *PD-L1*, *VISTA* and *TIM3* might reflect the necessity of the neoplastic cells to compensate the high immunologic visibility through the mechanism of aberrant activation of immune checkpoint genes that counteracts the cytotoxic effects of the immune response [61].

In summary, we identified the new candidate genes for LUSC and a cancer gene expression signature that proved to be an effective classifier of LUSC tumors and a biomarker for predicting survival outcome of LUSC patients. Overall, the identified novel patterns of clonal evolution, mutational landscapes and expression signatures of LUSC could contribute to the development of new LUSC therapeutic strategies.

## MATERIALS AND METHODS

### Mouse model and lung tissue collection

In this study, the NTCU induced NIH Swiss mouse lung squamous cell carcinoma (LUSC) model was used as previously reported [12, 62]. NTCU was purchased from Toronto Research Chemicals, Inc. All animal experiments were conducted with the approval of the Institutional Animal Care and Use Committee of the Medical College of Wisconsin (MCW). For bulk sequencing, lung tumor tissues were obtained from NTCU induced LUSC tumor-bearing NIH Swiss mice (*n* = 16) and normal lung tissues were obtained from control healthy NIH Swiss mice without NTCU treatment (*n* = 8). For single-cell RNA-sequencing (scRNA-seq), two LUSC tumors from another two NIH Swiss mice independent of the 16 mice subjected to the bulk RNA-seq were used. NTCU treatment caused the development of LUSC tumors within 24 to 26 weeks [63]. In this study, mice LUSC tumor tissues were obtained 28 weeks after the initial treatment of NTCU when mice were euthanized by CO_2_ asphyxiation.

### Bulk DNA/RNA extraction and sequencing

For the bulk exome-sequencing (exome-seq) and RNA-sequencing (RNA-seq), both the DNA and RNA samples from each lung tissue sample (SCC tumor or normal) were isolated simultaneously using the AllPrep DNA/RNA Mini Kit (Qiagen Inc., CA). Whole exome sequencing (WES) was conducted on 16 lung SCC tumors and 8 normal lung tissue samples. Detailed WES procedures can be seen in our previous publication [64]. Briefly, whole exome capture was carried out using the protocol for Agilent’s SureSelectXT Mouse All Exon Kit. The exome-seq libraries were sequenced for 100 bp paired-end reads by the MCW Human and Molecular Genetics Center (HMGC) Sequencing Core using HiSeq 2500 platforms (Illumina, San Diego, CA). Each sample was sequenced at a mean depth of about 133 X. Sequence short reads were aligned to a reference genome (NCBI mouse genome assembly mm9) using the BWA (Burrows-Wheeler Aligner) program [65]. Each alignment was assigned a mapping quality score by BWA [65], which is the Phred-scaled probability that the alignment is incorrect. Reads with low mapping quality scores (<5) were removed to reduce false positive rate. The PCR duplicates were detected and removed by the Picard program. We then performed local realignment of the BWA-aligned reads using the Genome Analysis Toolkit (GATK) [66]. VarScan 2 [67] was used to call somatic variants based on the local realignment results comparing each tumor with the eight normal lung samples. Default parameters in VarScan 2 were used. The lists of shared SNVs/indels were then annotated using ANNOVAR [68]. Single nucleotide polymorphisms (SNPs) were filtered using the mouse dbSNP VCF file (mm9_snp128). For RNA-seq, the quality of the total RNA samples obtained was very high, with RIN (RNA integrity number) values in the range of 9–10. The TruSeq RNA Library Preparation Kit v2 (Illumina Inc., CA) was used to construct the RNA-seq libraries. The sequencing of the RNA-seq library samples was performed in MCW-HMGC Sequencing Core using HiSeq 2500 platforms. The reads generated were pair-end with 100 nucleotides in length. The qualities of the RNA-seq reads were analyzed using the FastQC program (http://www.bioinformatics.babraham.ac.uk/projects/fastqc/). The coverage ranged from 15 million to 32 million reads per RNA-seq sample. The quality scores of >95.3% of all the bases of each sample are >30, averaging around 40. The pre-processed sample RNA-seq reads were aligned to the mm9 mouse genome using Bowtie-TopHat [version 2.0.4 [69, 70]]. Gene-level read counts were obtained using the htseq-count Python script (http://www-huber.embl.de/users/anders/HTSeq/) in the “union” mode. Further differential gene expression analysis was conducted using edgeR software [71]. FDR (False discovery rate) corrected *P*-values of less than 0.05 were used as criteria for significantly regulated genes.

### Single-cell isolation and RNA sequencing

Single-cell suspensions were prepared from two mouse LUSC tumor tissue samples using a mouse Tumor Dissociation kit (Miltenyi Biotec Inc., CA). For single-cell analysis cell capture, lysis, reverse transcription, and cDNA amplification were performed on the C1 integrated fluidic circuit (IFC) for mRNA-seq on a Fluidigm C1 Single-Cell Auto Prep System following the manufacturer’s protocol (Fluidigm Corporation, CA). Medium-sized C1 mRNA-Seq chips (10–17 μm) were used to capture single cells from each of the two tumors. The C1 Auto Prep System captures the dissociated single cells and the whole-transcriptome amplified cDNA was prepared on chip using the SMARTer Ultra Low RNA kit from Illumina (Clontech). Cells captured are manually inspected as a quality control measure to remove empty well, doublets, or debris-containing wells. cDNA from cells are checked by Qubit dsDNA HS Assay Kit (Thermo Fisher Scientific). Single-cell libraries were constructed with the use of the Illumina Nextera XT DNA Sample Preparation kit with 96 dual barcoded indices and were multiplexed and sequenced to a depth of 2–4 million reads (HiSeq 2500; Illumina) using 50-bp single-end sequencing. The program RSEM [72] was utilized to quantify transcript expression. Differential expression analysis was conducted on RSEM derived TPM (Transcripts Per Kilobase Million) values using the software AltAnalyzer [73]. FDR (False discovery rate) corrected *P*-values of less than 0.05 were used as criteria for significantly regulated genes.

### Clonal mutation, pathway enrichment, survival and association analyses

The bioinformatics tools – SciClone [74] and clonevol package (https://github.com/hdng/clonevol) were used to identify the clonal structures of each of the 16 mouse LUSC tumors. Single-cell gene mutations were analyzed using the rnaseqmut program (https://github.com/davidliwei/rnaseqmut/blob/master/README) based on scRNA-seq data. The software SCITE [75] was used to analyze the orders of the sequential mutational events in the two LUSC tumors subjected to scRNA-seq. Pathway enrichment analysis of the G80 module cancer genes was performed by using ConsensusPathDB software (http://cpdb.molgen.mpg.de/). Four published lung cancer gene expression data sets [18–20, 56] were analyzed using the program SurvExpress [76] to test whether the G80 cancer gene module was associated with overall survival of the patients. In order to test whether these mutated genes have potential clinical application, the associations between mutational status of the identified LUSC genes and markers of immune response were analyzed by using the set of 176 TCGA LUSC tumor samples with gene mutation data and expression data available through NCI GDC Data Portal (https://portal.gdc.cancer.gov/). The association test between mutation genotypes and immune checkpoint gene expression in LUSC was performed using the t test implemented in the computing environment R (R Development Core Team, 2005). FDR (False discovery rate) corrected *p*-values of less than 0.05 were used as criteria for significant association. Plots of mutations were generated using the “oncoPrint” function provided by the R package – ComplexHeatmap [77] and gene expression heatmaps were generated using the R package – heatmap3 (https://cran.r-project.org/web/packages/heatmap3/).

## SUPPLEMENTARY MATERIALS FIGURES AND TABLES






