# Supplementary material for: Novel mutational landscapes and expression signatures of lung squamous cell carcinoma
Source: Oncotarget. 2017 Dec 27;9(7):7424–41. doi: 10.18632/oncotarget.23716 (PMC5800913; doi:10.18632/oncotarget.23716)
Supplement: Supplementary file 1 [file oncotarget-09-7424-s001.pdf]

Novel mutational landscapes and expression signatures of lung squamous cell carcinoma

SUPPLEMENTARY MATERIALS

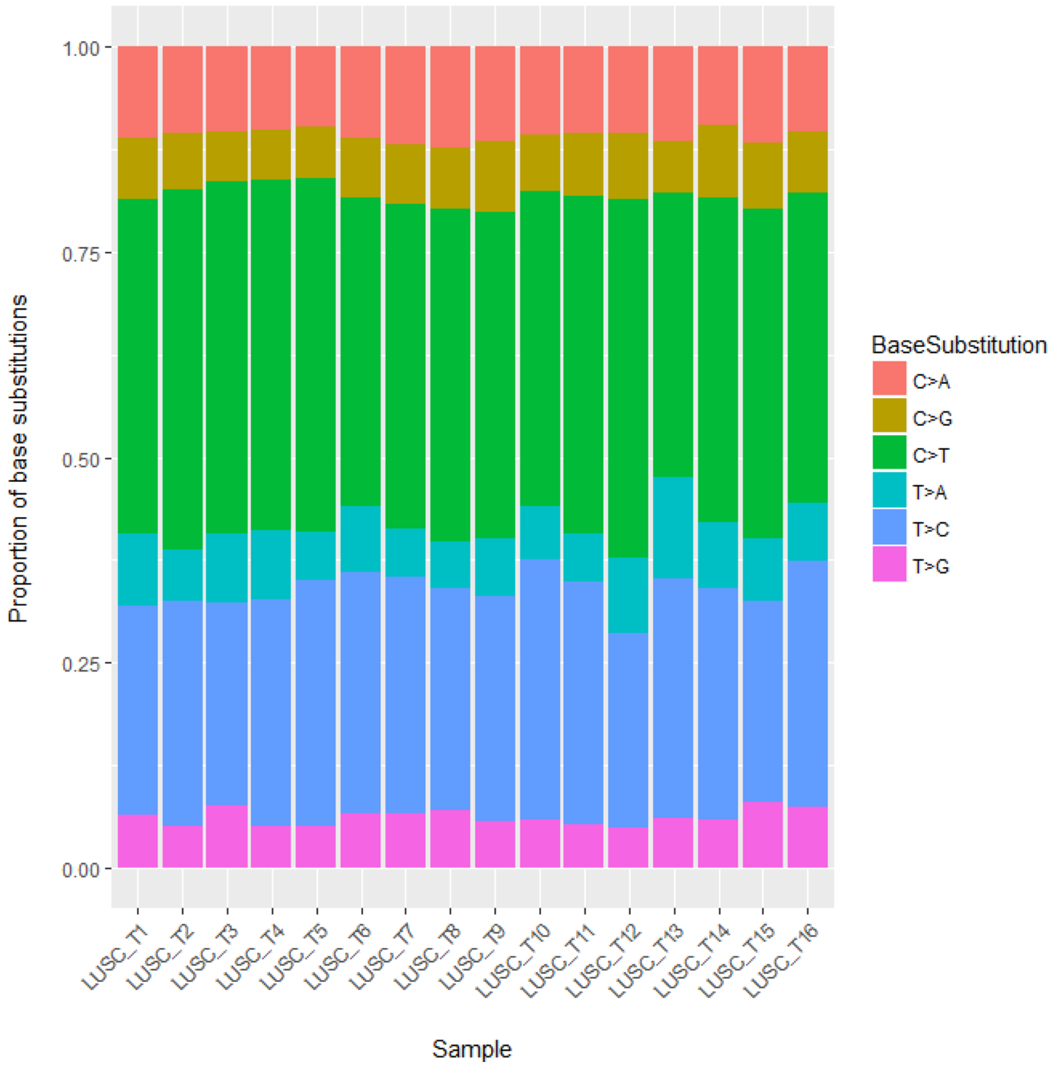

Supplementary Figure 1: Spectrum of base substitutions based in the 16 mouse LUSCs. The most common substitutions were C>T transversions. In addition, it can be seen that there is no difference in the proportion of each mutation category between the mouse LUSC tumors with wild-type *Trp53* (LUSC_T1 to LUSC_T8) and those with the *Trp53* p.A135V germline mutation (LUSC_T9 to LUSC_T16), suggesting that the somatic coding mutations were mainly driven by the exposure to NTCU rather than the *Trp53* p.A135V germline mutation.

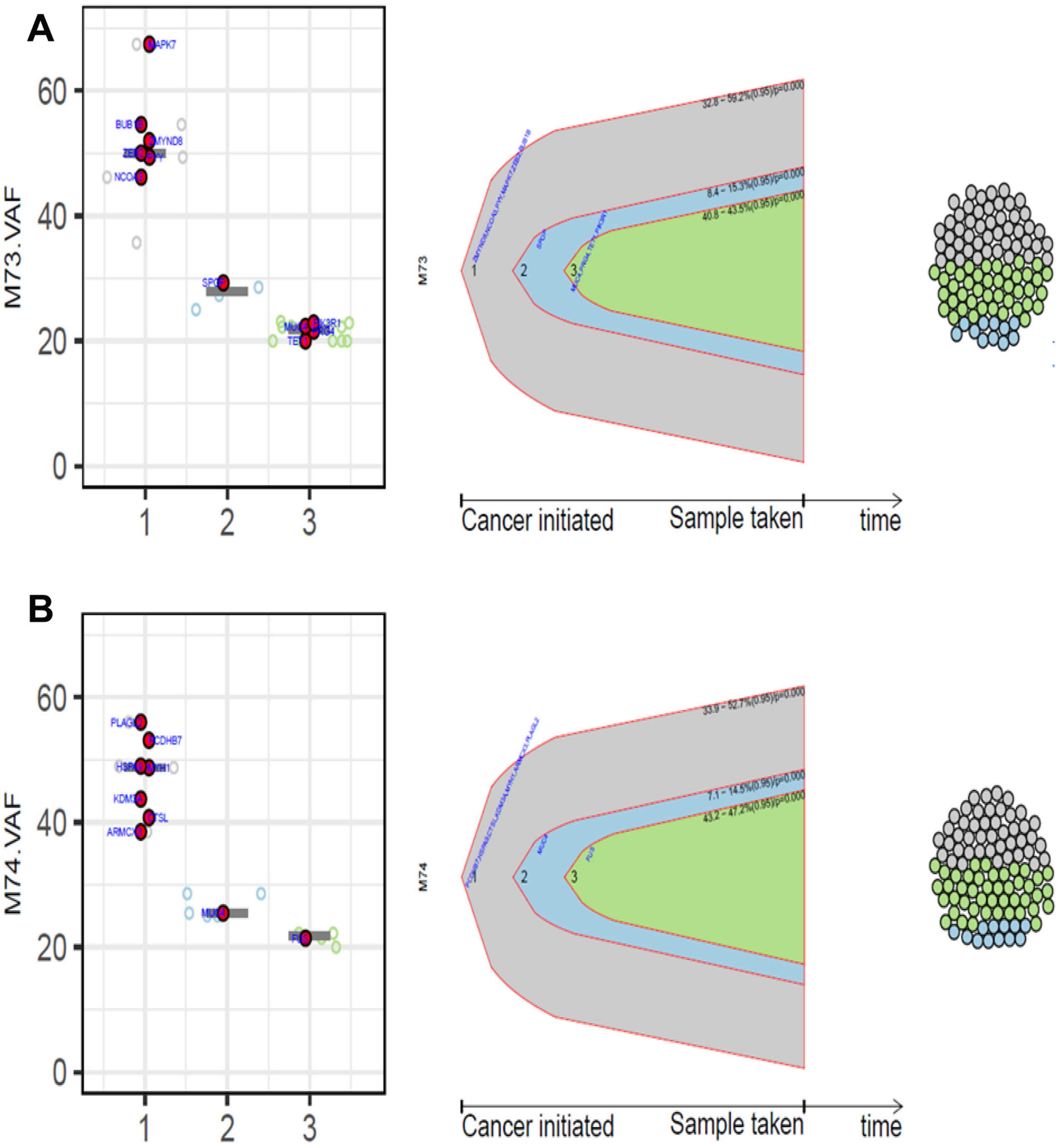
A
M73.VAF
60
40
20
0
1
2
3
M73
Cancer initiated
Sample taken
time
B
M74.VAF
60
40
20
0
1
2
3
M74
Cancer initiated
Sample taken
time

C

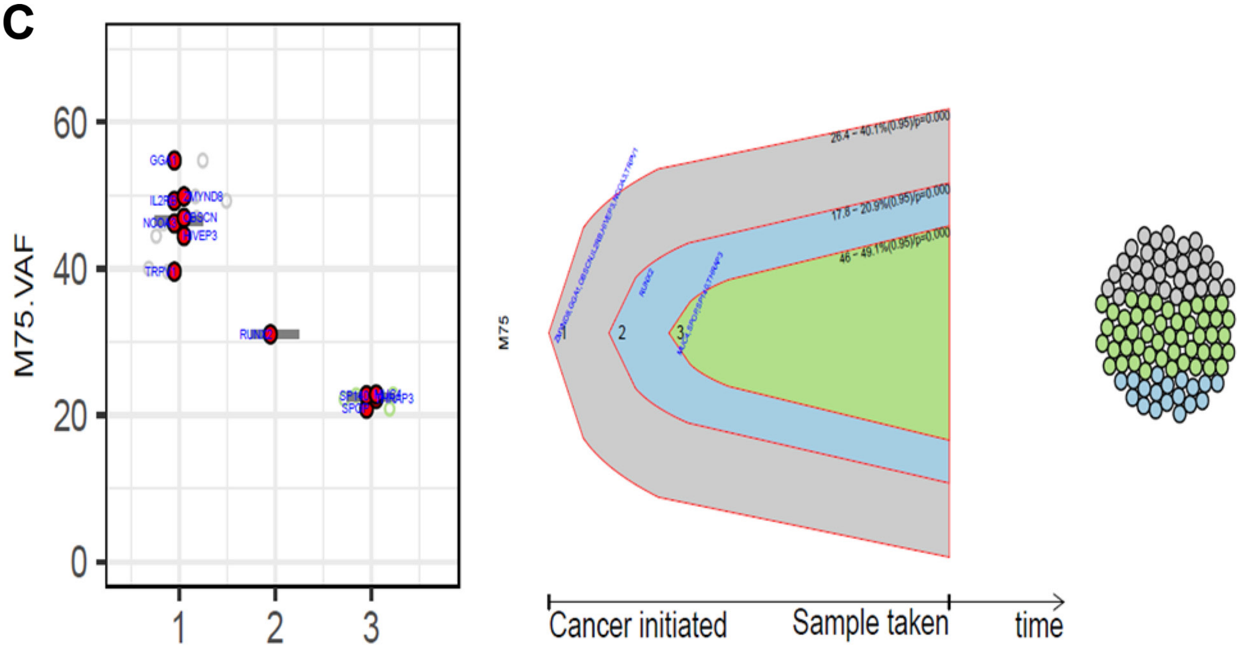
M75.VAF
0
20
40
60
1
2
3
M75
Cancer initiated
Sample taken
time

D

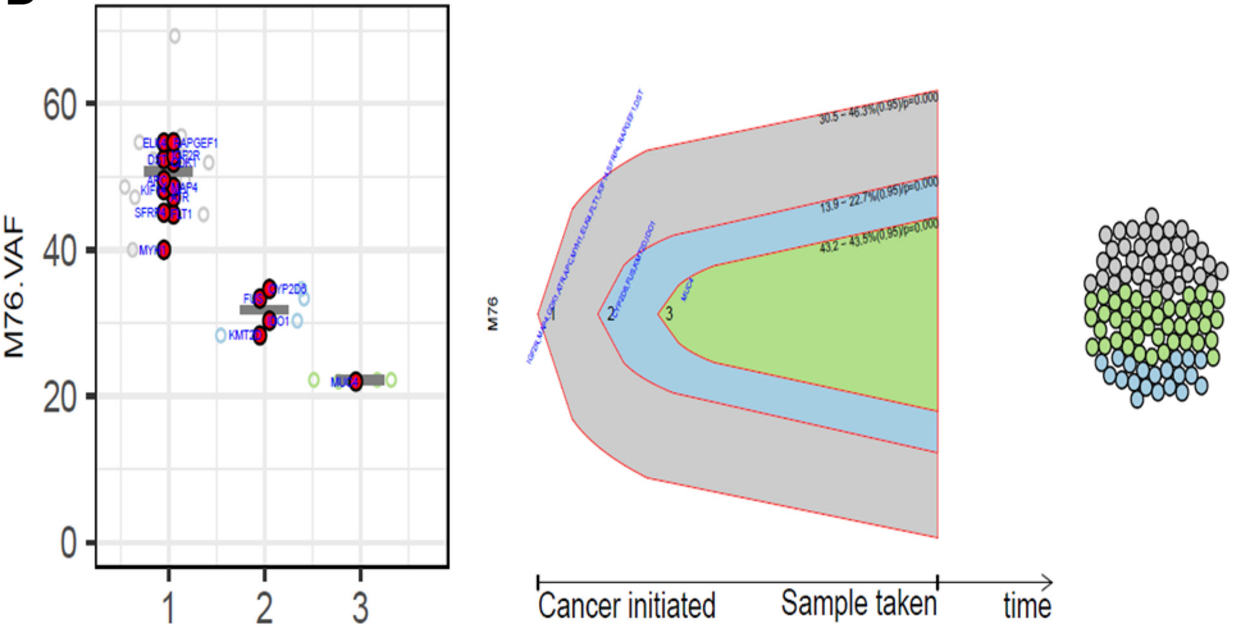
M76.VAF
0
20
40
60
1
2
3
M76
Cancer initiated
Sample taken
time

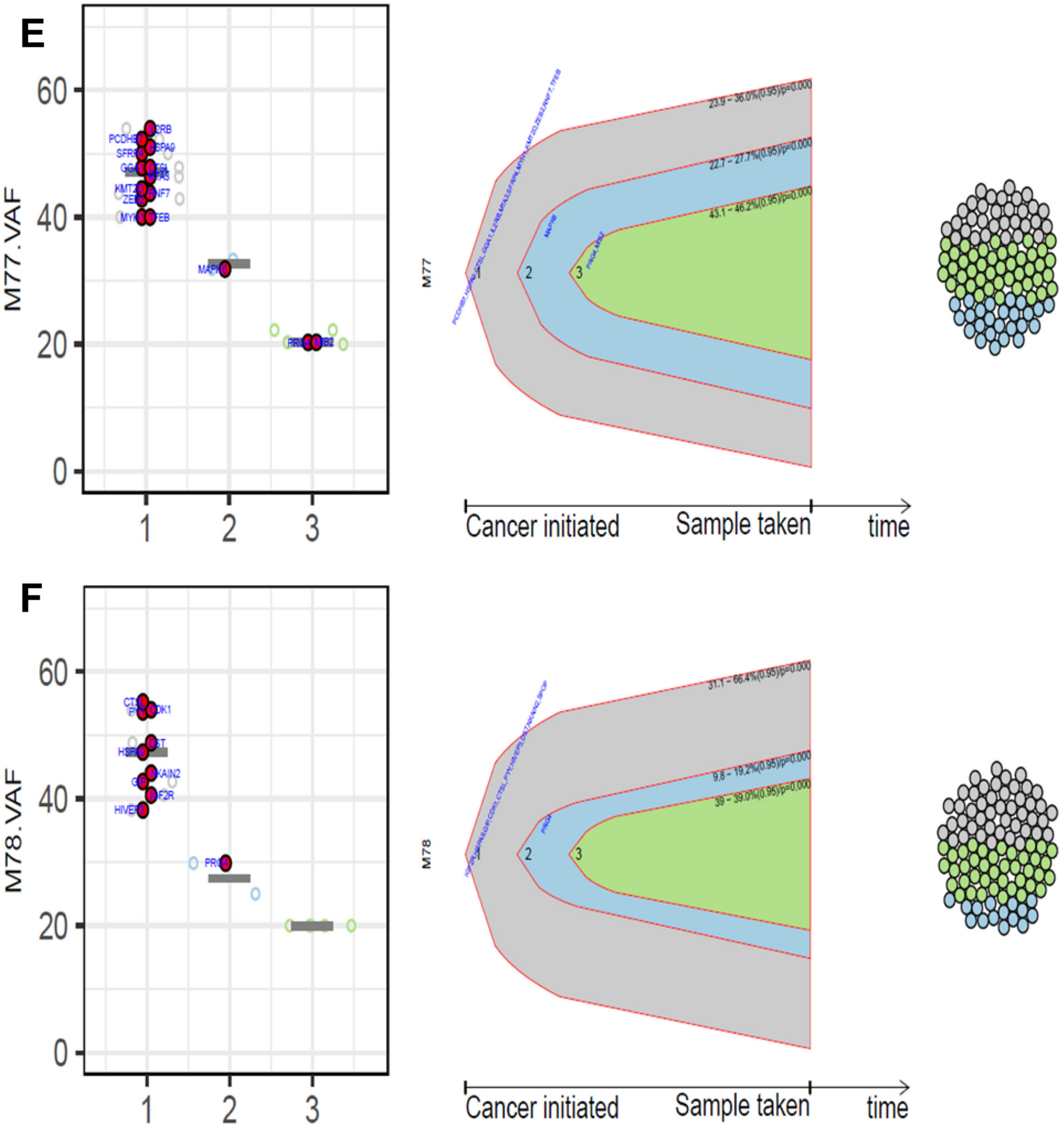
E
M77.VAF
60
40
20
0
1
2
3
M77
Cancer initiated
Sample taken
time
F
M78.VAF
60
40
20
0
1
2
3
M78
Cancer initiated
Sample taken
time

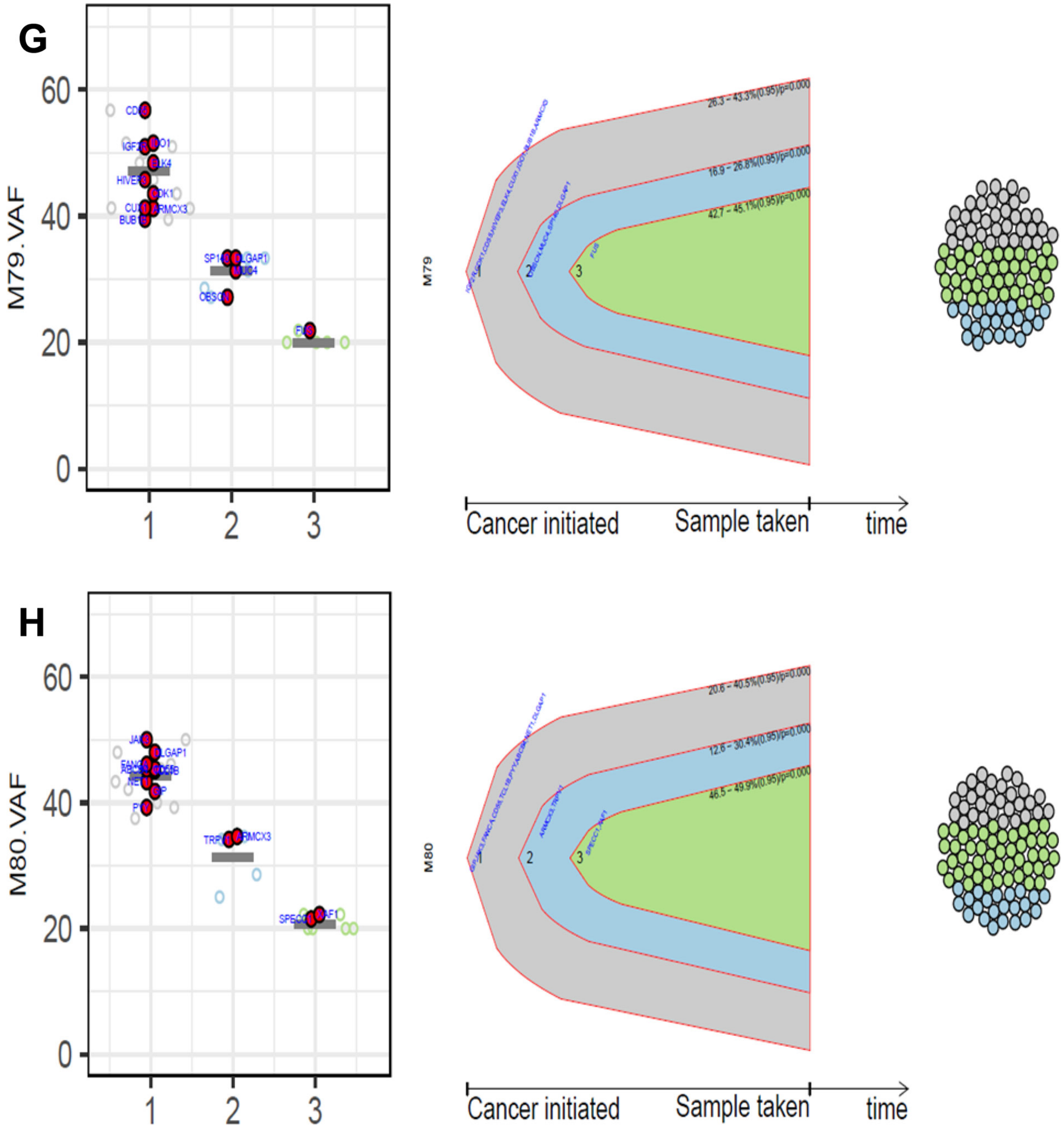
G
M79.VAF
60
40
20
0
1
2
3
Cancer initiated
Sample taken
time
H
M80.VAF
60
40
20
0
1
2
3
Cancer initiated
Sample taken
time

I

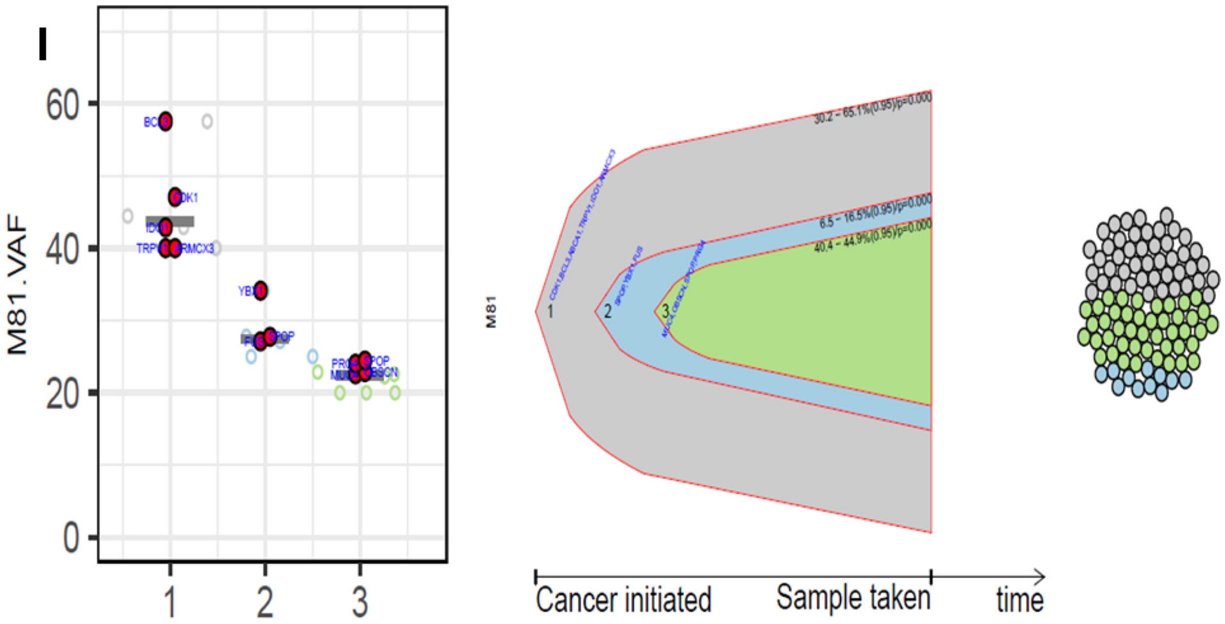

M81. VAF
0
20
40
60
1
2
3
ARMCX3
M81
1
2
3
30.2 - 65.1%(0.95)/p=0.000
6.5 - 16.5%(0.95)/p=0.000
40.4 - 44.9%(0.95)/p=0.000
Cancer initiated
Sample taken
time

J

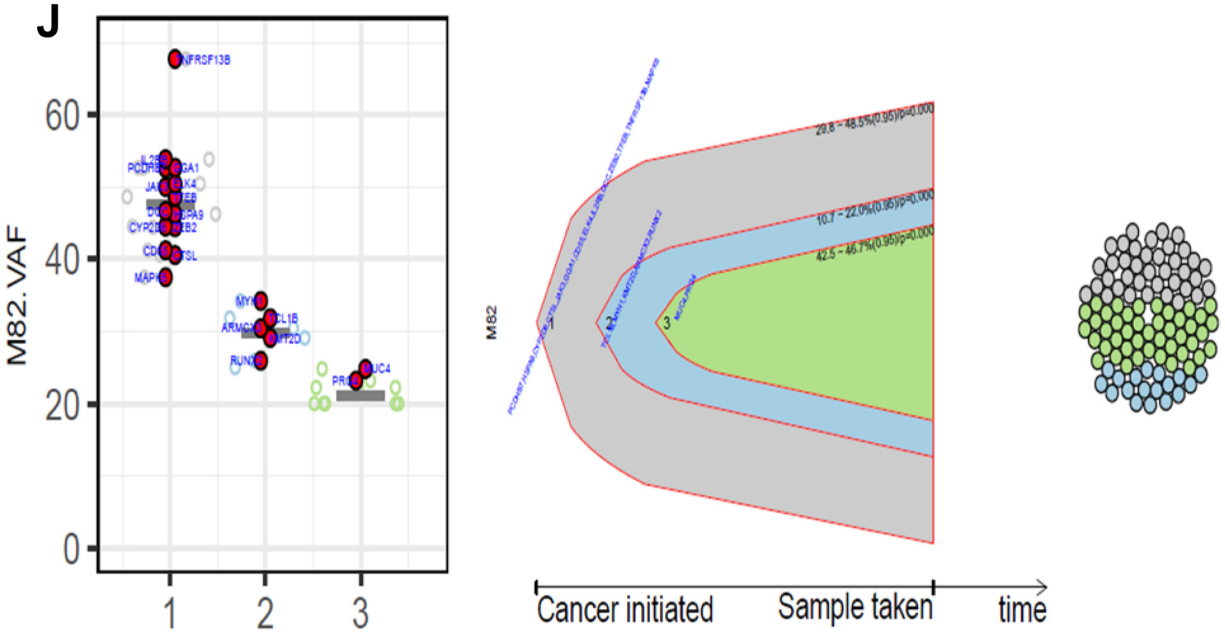

M82. VAF
0
20
40
60
1
2
3
TNFRSF13B
M82
1
2
3
29.8 - 48.5%(0.95)/p=0.000
10.7 - 22.0%(0.95)/p=0.000
42.5 - 46.7%(0.95)/p=0.000
Cancer initiated
Sample taken
time

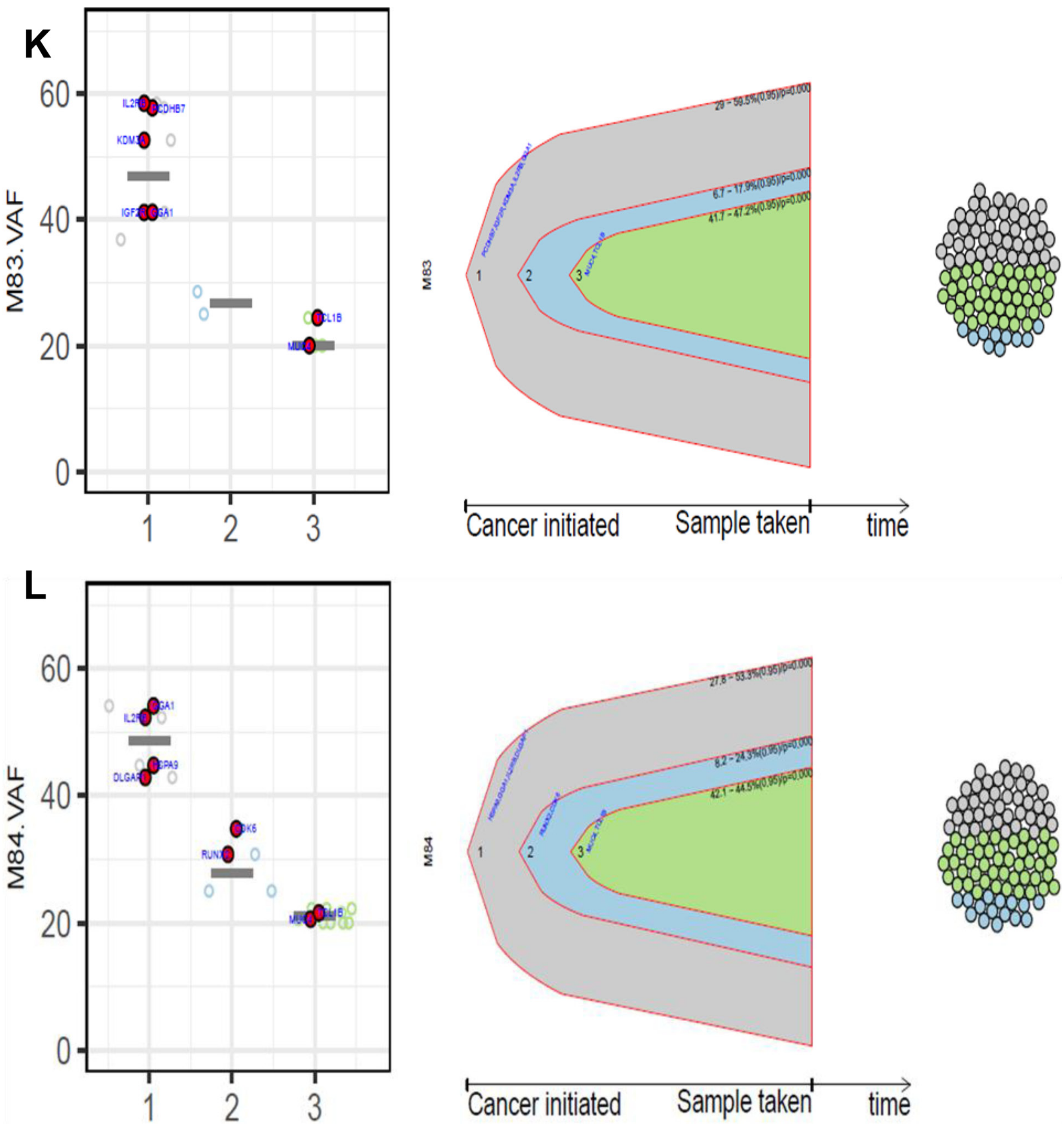
K
M83. VAF
60
40
20
0
1
2
3
M83
1
2
3
Cancer initiated
Sample taken
time
L
M84. VAF
60
40
20
0
1
2
3
M84
1
2
3
Cancer initiated
Sample taken
time

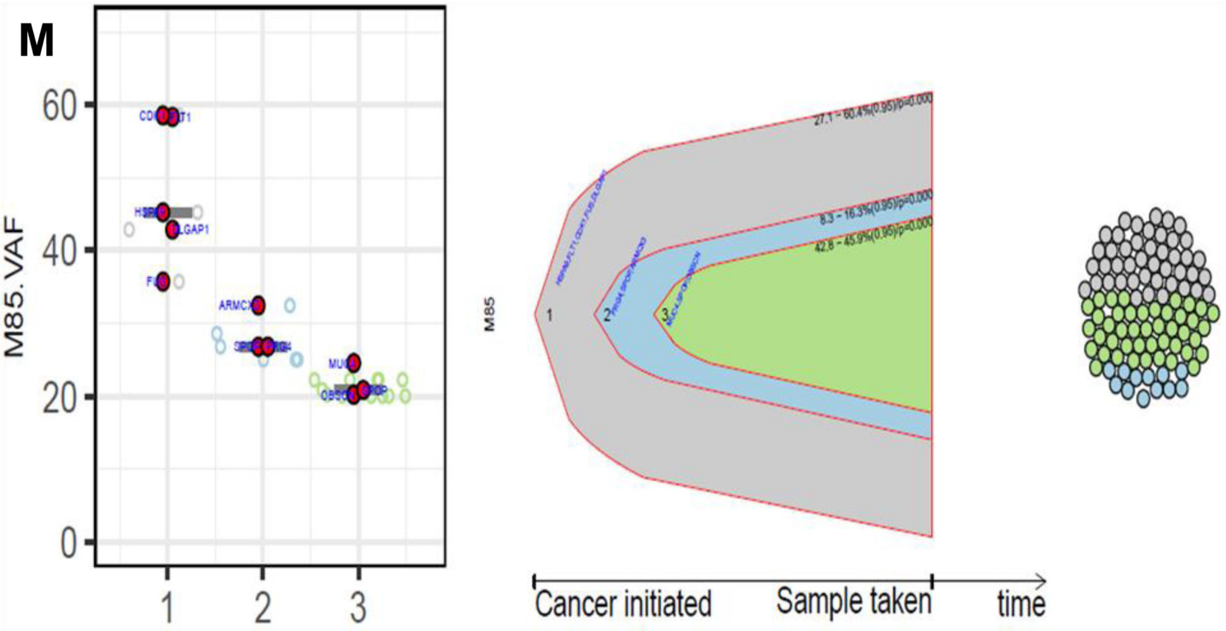
M
M85. VAF
60
40
20
0
1
2
3
M85
Cancer initiated
Sample taken
time

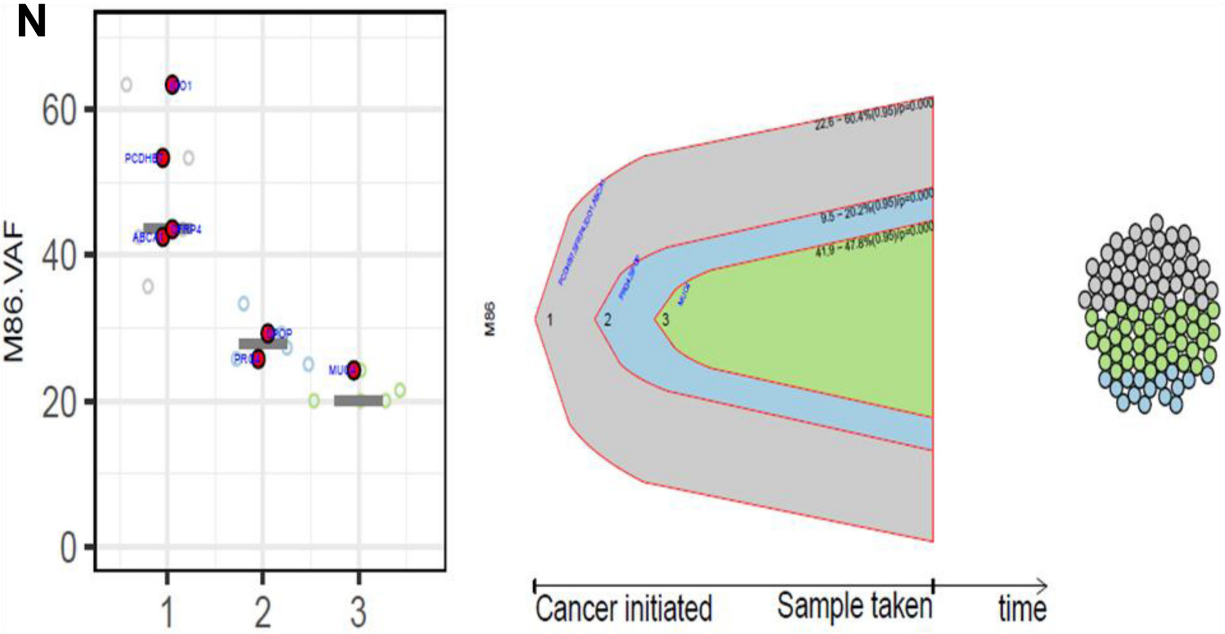
N
M86. VAF
60
40
20
0
1
2
3
M86
Cancer initiated
Sample taken
time

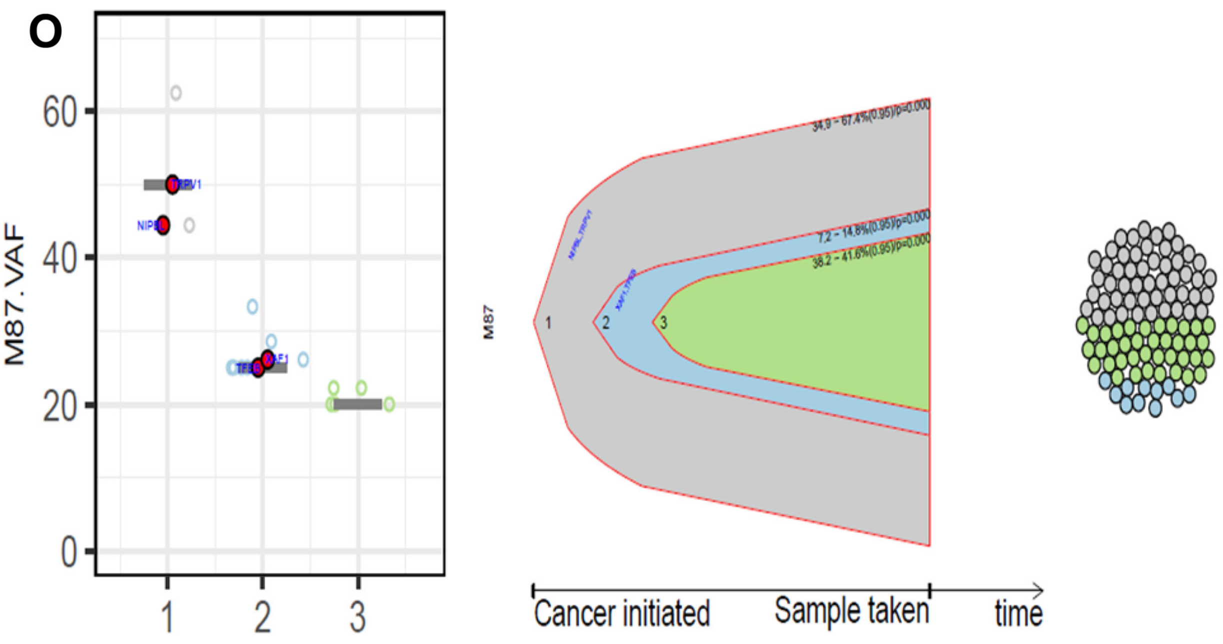

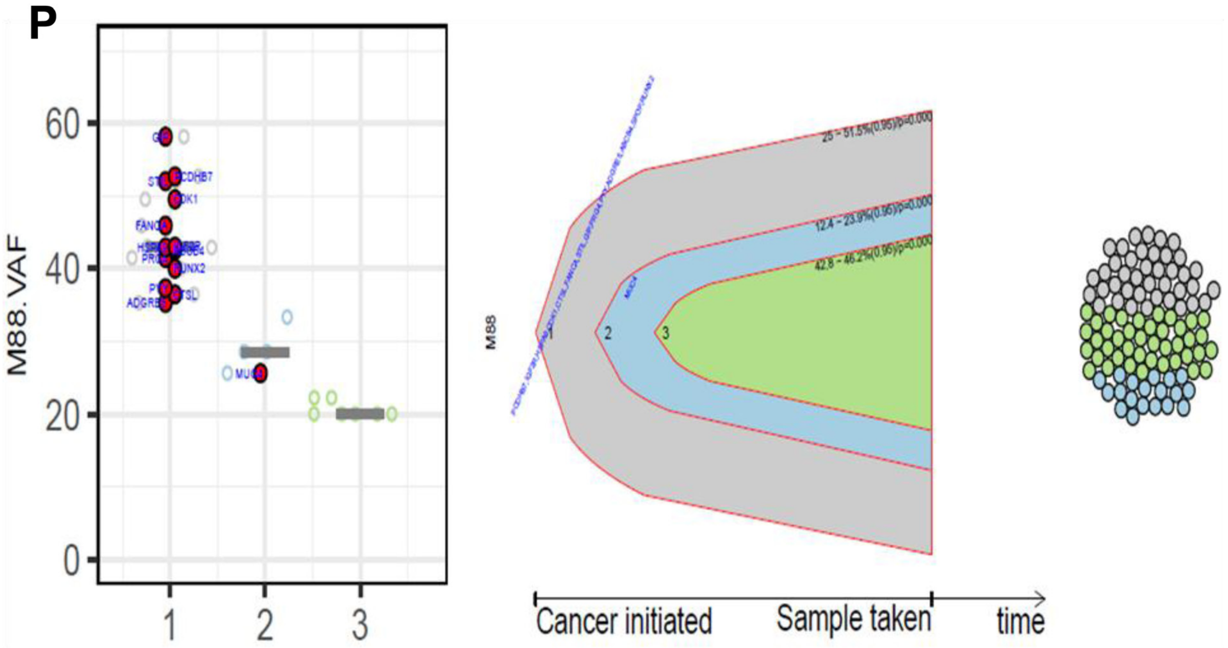

Supplementary Figure 2: Clonal analysis of 16 mouse lung squamous carcinoma tumors – LUSC_T1 to LUSC_T16. (**A**) Mouse LUSC_T1 tumor: the cancer genes harboring the clonal mutations include *MAPK7, NCOA7, BUB1B, ZEB2, ZMYND8, PYY.* (**B**) Mouse LUSC_T2 tumor: the cancer genes harboring the clonal mutations include *PCDHB7, HSPA9, CTSL, KDM3A, MYH1, ARMCX3, PLAGL2*. (**C**) Mouse LUSC_T3 tumor: the cancer genes harboring the clonal mutations include *ZMYND8, GGA1, OBSCN, IL2RB, HIVEP3, NCOA7, TRPV1*. (**D**) Mouse LUSC_T4 tumor: the cancer genes harboring the clonal mutations include *IGF2R, MAP4, CDK1, ATR, APC, MYH1, ELK4, FLT1, KIF14, SFRP4, RAPGEF1, DST*. (**E**) Mouse LUSC_T5 tumor: the cancer genes harboring the clonal mutations include *PCDHB7, HSPA9, CTSL, GGA1, IL2RB, MTA3, SFRP4, MYH1, KMT2D, ZEB2, RNF7, TFEB*. (**F**) Mouse LUSC_T6 tumor: the cancer genes harboring the clonal mutations include *IGF2R, HSPA9, GIP, CDK1, CTSL, PYY, HIVEP3, DST, NKAIN2, SPOP*. (**G**) Mouse LUSC_T7 tumor: the cancer genes harboring the clonal mutations include *IGF2R, CDK1, CD55, HIVEP3, ELK4, CUX1, IDO1, BUB1B, ARMCX3*. (**H**) *Mouse LUSC_T8* tumor: the cancer genes harboring the clonal mutations include *GIP, JAK3, FANCA, CD55, TCL1B, PYY, ABCB4, NET1, DLGAP1*. (**I**) Mouse LUSC_T9 tumor: the cancer genes harboring the clonal mutations include *CDK1, BCL3, ABCA1, TRPV1, IDO1, ARMCX3*. (**J**) Mouse LUSC_T10 tumor: the cancer genes harboring the clonal mutations include *HSPA9, CYP2D6, CTSL, JAK3, GGA1, CD55, ELK4, IL2RB, DCC, ZEB2, TFEB, TNFRSF13B, MAPK6*. (**K**) Mouse LUSC_T11 tumor: the cancer genes harboring the clonal mutations include *PCDHB7, IGF2R, KDM3A, IL2RB, GGA1*. (**L**) Mouse LUSC_T12 tumor: the cancer genes harboring the clonal mutations include *HSPA9, GGA1, IL2RB, DLGAP1*. (**M**) Mouse LUSC_T13 tumor: the cancer genes harboring the clonal mutations include *HSPA9, FLT1, CDK1, FUS, DLGAP1*. (**N**) Mouse LUSC_T14 tumor: the cancer genes harboring the clonal mutations include *PCDHB7, SFRP4, IDO1, ABCA1*. (**O**) Mouse LUSC_T15 tumor: the cancer genes harboring the clonal mutations include *NIPBL* and *TRPV1*. (**P**) Mouse LUSC_T16 tumor: the cancer genes harboring the clonal mutations include *IGF2R, HSPA9, CDK1, CTSL, FANCA, STIL, GIP, PRG4, PYY, ADGRE5, ABCB4, SPOP, RUNX2*.

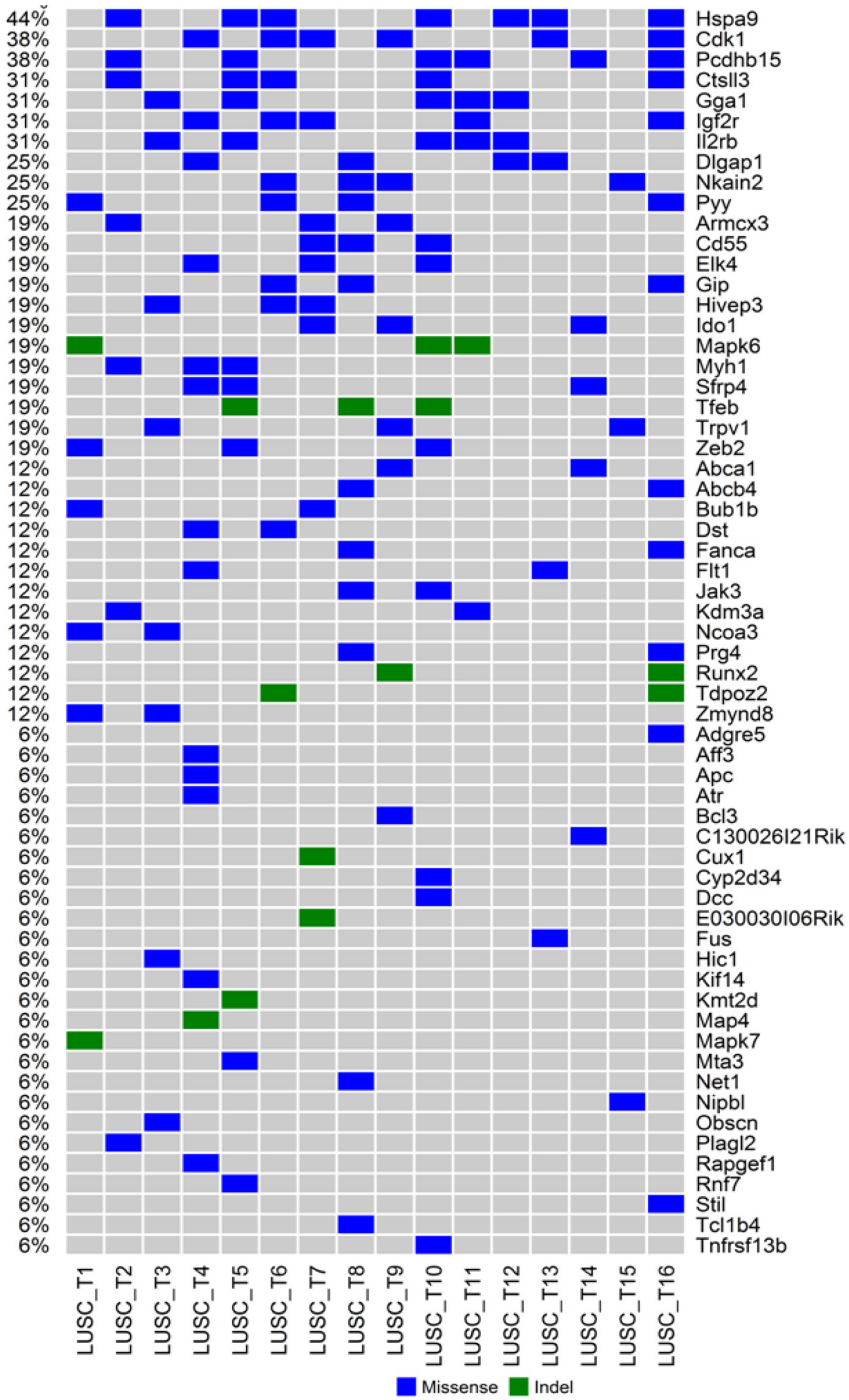

Supplementary Figure 3: The clonal mutation spectrum across the 16 mice LUSC tumors. There were nine most frequent clonal mutations identified in at least 4 of 16 LUSC tumors, including *Hspa9*: A651S (7), *Cdk1*: S39I (6), *Pcdhb15*: R461C (6), *Ctsll3*: P329S (5), *Gga1*: D358N (5), *Il2rb*: R475S (5), *Dlgap1*: A329T (4), *Nkain2*: V67L (4), and *Pyy*: P42L (4). Further details can be seen in Supplementary Table 3.

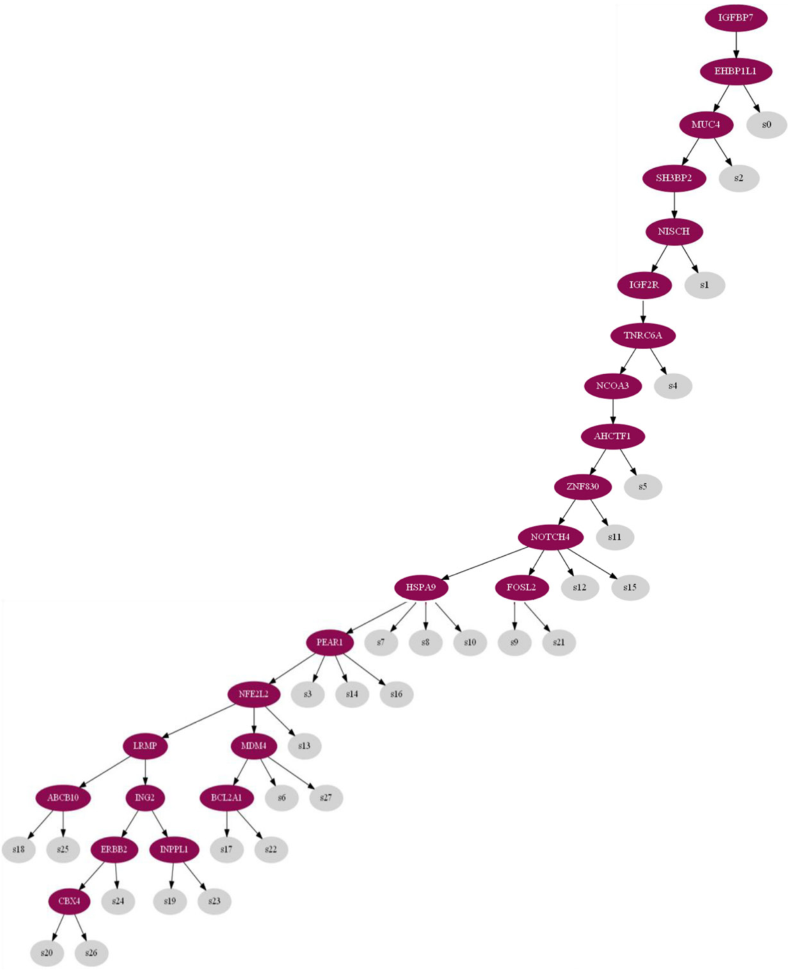

Supplementary Figure 4: The evolution history based on the 23 somatic mutated genes identified from scRNA-seq data for the first mouse lung SCC tumor: LUSC1. Cell IDs were marked as s0 to s27, corresponding to the single tumor cells of LUSC1_sc1 to LUSC1_sc28.

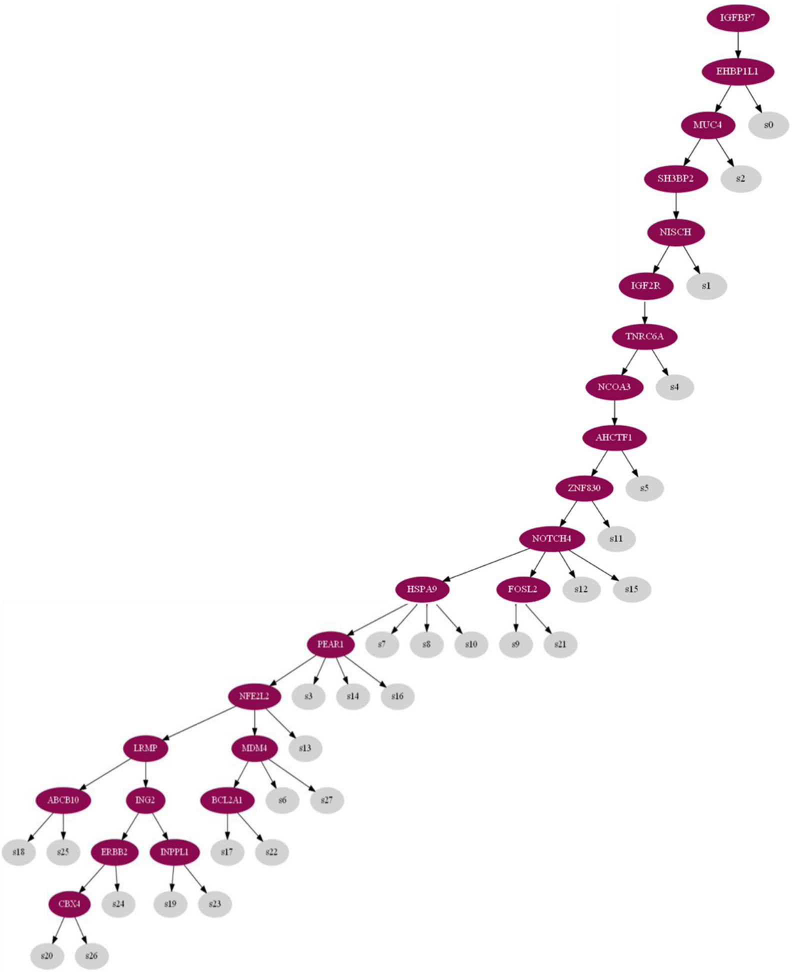

Supplementary Figure 5: The evolution history based on the 15 somatic mutated genes identified from scRNA-seq data for the second mouse lung SCC tumor: LUSC2. Cell IDs were marked as s0 to s19, corresponding to the single tumor cells of LUSC2_sc1 to LUSC2_sc20.

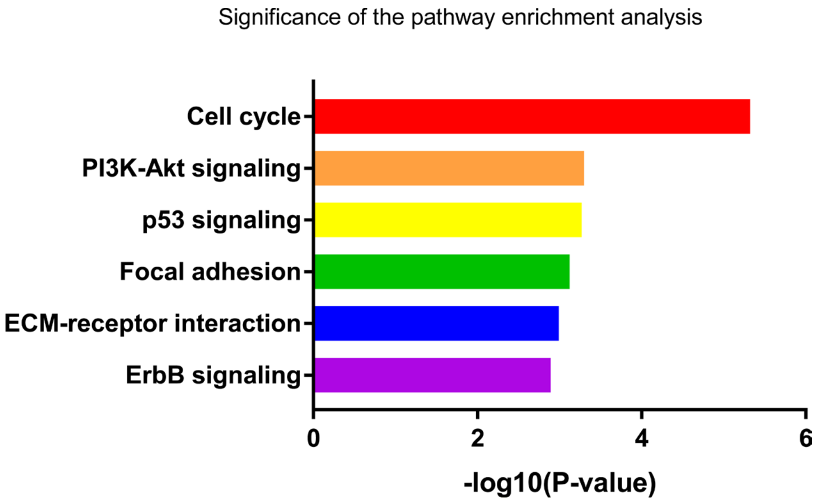

Supplementary Figure 6: The G80 module member genes were significantly enriched in the six cancer related molecular pathways.

A

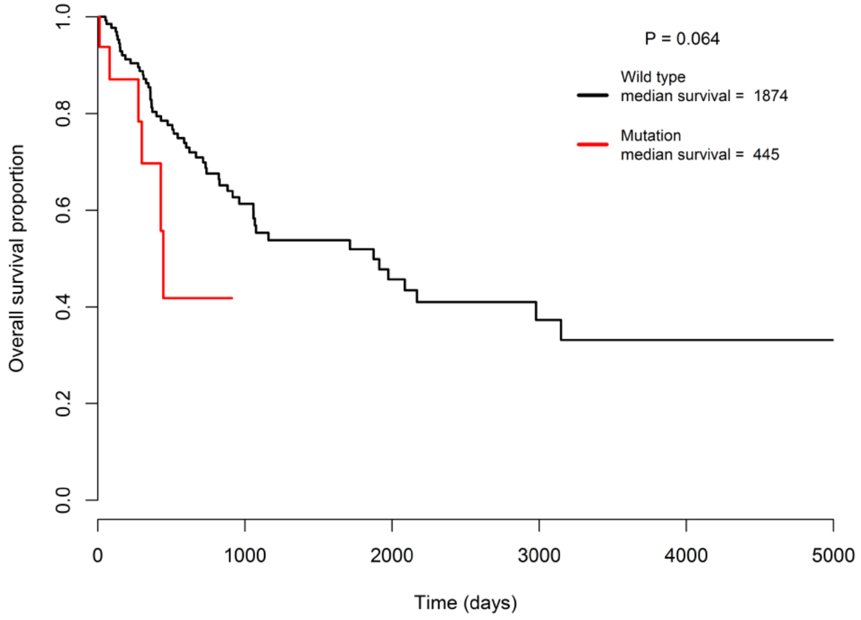

B

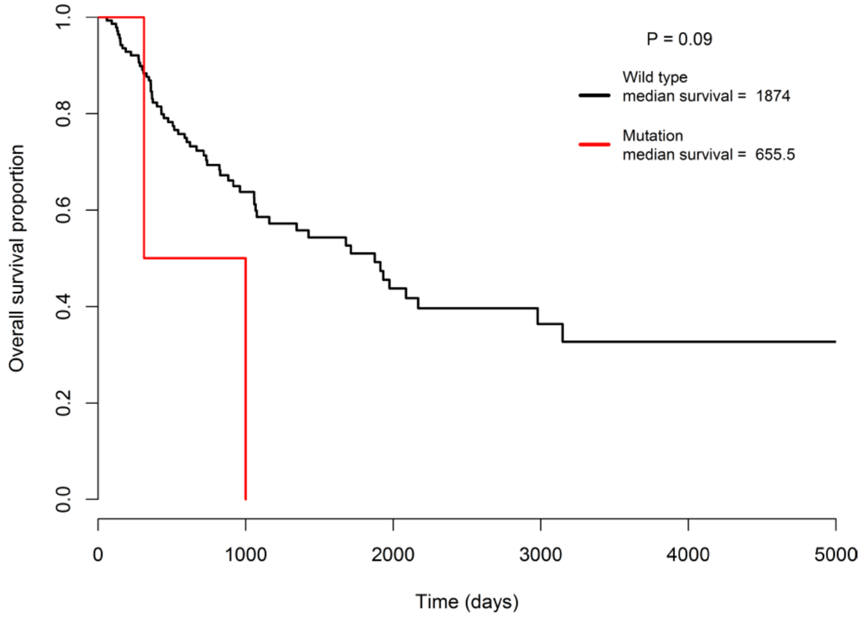

Supplementary Figure 7: Survival outcome analysis based on the mutation status of two LUSC oncogenes. There were trends toward significance for the associations of worse overall survival outcome with *NFE2L2* and *FLT1* mutations (*P* values = 0.06 and 0.09, respectively).

Supplementary Table 1: Whole-exome sequencing quality control.

Normal lung tissues	Total reads	Proportion of reads in exon regions (%)	Mean exon coverage	% of bases covered at least 20 X
Normal1	148,305,725	48.4%	152.7	85.6%
Normal2	140,789,378	48.2%	143	85.6%
Normal3	137,008,160	47.9%	139.5	85.6%
Normal4	123,729,539	48.4%	126.5	85.1%
Normal5	125,221,430	48.9%	129.1	84.8%
Normal6	118,934,788	46.9%	116	84.1%
Normal7	113,745,101	47.3%	112.4	83.7%
Normal8	184,503,827	47.1%	179	86.2%
Average targeted reads coverage of 8 normal lung samples: 137 X	Range of targeted reads coverage of 8 normal lung samples: 112 X -179 X			

Lung squamouse cell carcinomas	Total reads	Proportion of reads in exon regions (%)	Mean exon coverage	% of bases covered at least 20 X
LUSC_T1	115,623,278	46.6%	113.2	84.5%
LUSC_T2	143,468,652	47.3%	144.4	85.4%
LUSC_T3	146,429,531	47.0%	145.7	86.1%
LUSC_T4	117,332,091	46.3%	113.7	84.9%
LUSC_T5	152,956,054	47.8%	156.3	85.6%
LUSC_T6	120,838,889	49.5%	127.4	85.3%
LUSC_T7	139,534,885	48.9%	144.2	86.1%
LUSC_T8	122,729,417	49.9%	130	84.5%
LUSC_T9	137,098,819	49.5%	144.9	85.4%
LUSC_T10	156,753,606	49.4%	164.9	86.0%
LUSC_T11	131,113,699	48.8%	136.4	85.0%
LUSC_T12	106,097,197	48.7%	109.2	84.2%
LUSC_T13	108,925,874	49.4%	110.2	83.1%
LUSC_T14	93,847,098	49.4%	97.7	83.1%
LUSC_T15	96,740,881	49.7%	101.7	82.9%
LUSC_T16	113,586,184	49.6%	118.7	84.5%
Average targeted reads coverage of 16 LUSC tumor samples: 129 X	Range of targeted reads coverage of 16 LUSC tumor samples: 98 X - 165 X			

Exon reads coverages among 8 normal lung tissue samples and 16 LUSC tumor samples

Supplementary Table 2: The list of 5664 somatic coding mutations across the 16 mice LUSC tumors, which consisted of 2885 missense, 106 nonsense, 2426 silent mutations, 167 small insertions and deletions (indels) and 80 alterations residing in exon-exon boundaries. See Supplementary_Table_2

Supplementary Table 3: The clonal mutations identified across the 16 mouse LUSC tumors. See Supplementary_Table_3

Supplementary Table 4: The details of the cancer gene mutations identified in the two mice LUSC tumors subjected to scRNA-seq

Mouse gene	Chr	Start	Ref	Alt	Amino acid change	Tumor
Igfbp7	chr5	77836906	C	G	p.R45P	LUSC1
Muc4	chr16	32776908	A	G	p.M3179V	LUSC1
Ncoa3	chr2	165880571	G	A	p.S594N	LUSC1
Hspa9	chr18	35098249	C	A	p.A651S	LUSC1
Igf2r	chr17	12876657	T	G	p.R2457S	LUSC1
Nfe2l2	chr2	75514233	C	G	p.E527Q	LUSC1
Ahctf1	chr1	181683155	G	A	p.T1871I	LUSC1
Notch4	chr17	34721987	G	A	p.G1549S	LUSC1
Sh3bp2	chr5	34901808	T	C	p.S293P	LUSC1
Nisch	chr14	31984857	A	C	p.V1560G	LUSC1
Ehbp1l1	chr19	5719958	G	A	p.P439L	LUSC1
Tnrc6a	chr7	130314100	C	T	p.A533V	LUSC1
Zfp830	chr11	82578439	G	A	p.R189K	LUSC1
Pear1	chr3	87554196	G	A	p.P993S	LUSC1
Lrmp	chr6	145116556	C	A	p.A310D	LUSC1
Mdm4	chr1	134888279	G	A	p.A476V	LUSC1
Abcb10	chr8	126506478	C	G	p.C79S	LUSC1
Bcl2a1d	chr9	88626151	C	T	p.R136Q	LUSC1
Cbx4	chr11	118943450	A	G	p.S138P	LUSC1
Erbb2	chr11	98289242	G	A	p.V511I	LUSC1
Fosl2	chr5	32455267	A	C	p.D229A	LUSC1
Ing2	chr8	48759959	A	T	p.S26T	LUSC1
Inppl1	chr7	108978612	G	A	p.H525Y	LUSC1
Trp53	chr11	69401058	A	T	p.Q97L	LUSC2
Kmt2d	chr15	98670110	C	A	p.D5179Y	LUSC2
Cdk1	chr10	68808901	C	A	p.S39I	LUSC2
Keap1	chr9	21041687	A	G	p.M156T	LUSC2
Ppm1m	chr9	106101479	G	A	p.L16F	LUSC2
Dync1h1	chr12	111897370	A	G	p.T3919A	LUSC2
Myh9	chr15	77599352	A	C	p.V1405G	LUSC2
Rasgrp2	chr19	6413957	C	T	p.P562S	LUSC2
Slc19a1	chr10	76505074	C	G	p.R233G	LUSC2
Tbl3	chr17	24841403	G	A	p.T299I	LUSC2
Ltb	chr17	35332104	G	C	p.R91P	LUSC2
Swap70	chr7	117365328	A	G	p.R2G	LUSC2
Ing2	chr8	48759959	A	T	p.S26T	LUSC2
Mbd2	chr18	70728420	G	T	p.G117V	LUSC2
Pear1	chr3	87554196	G	A	p.P993S	LUSC2

Supplementary Table 5: Immune markers differentially expressed in lung squamous cell carcinomas based on mutation status of the significant genes

Immune marker	Gene	Fold change - Mutant vs Wt	*P*-value
PD-L1	HIVEP3	−4.6	2.83E-11
PD-L1	NKAIN2	−3.9	2.92E-11
PD-L1	RUNX2	−4.3	2.46E-09
PD-L1	MUC4	−3.0	2.31E-06
PD-L1	CUX1	−3.5	9.02E-06
PD-L1	NIPBL	−2.8	6.07E-05
PD-L1	PLAGL2	−3.4	0.000103
PD-L1	NFE2L2	1.9	0.018999
VISTA	KEAP1	−1.4	0.000573
VISTA	FANCA	−1.9	0.007671
VISTA	AFF3	−1.8	0.008345
VISTA	FLT1	1.5	0.019575
TIM3	RET	−1.9	4.17E-10
TIM3	FANCA	−2.2	0.000711
TIM3	ZMYND8	−1.8	0.028059
TIM3	DYNC1H1	1.3	0.026799
LAG3	CUX1	−3.0	6.88E-07
LAG3	FANCA	−2.4	0.000473
LAG3	NOTCH4	−2.0	0.027645

Supplementary Table 6: The G80 module member genes that were significantly enriched in the six cancer related molecular pathways

Pathway	*p*-value	*q*-value	Members_input_overlap
Cell cycle	4.77E-06	0.000196	CCNB1; CCNA2; BUB1B; PLK1; CCNE1; CDC20; CDK1
PI3K-Akt signaling	0.000501	0.005492	PGF; ITGB4; CCNE1; EGFR; EIF4EBP1; NGFR; TNC; SPP1
p53 signaling	0.000536	0.005492	CDK1; CCNB1; CCNE1; SERPINB5
Focal adhesion	0.000758	0.006213	PGF; ITGB4; EGFR; SPP1; TNC; PAK1
ECM-receptor interaction	0.001028	0.007025	SDC1; SPP1; ITGB4; TNC
ErbB signaling	0.001282	0.007508	CBLC; EIF4EBP1; EGFR; PAK1
