# Supplementary material for: Novel mutational landscapes and expression signatures of lung squamous cell carcinoma
Source: Oncotarget. 2017 Dec 27;9(7):7424–41. doi: 10.18632/oncotarget.23716 (PMC5800913; doi:10.18632/oncotarget.23716)
Supplement: Supplementary file 3 [file oncotarget-09-7424-s003.docx]

**Supplementary Table 3: The clonal mutations identified across the 16 mouse LUSC tumors**

| Chr | Position | Mouse gene | Human Symbol | Substitution | Consequence | Tumor |
| --- | --- | --- | --- | --- | --- | --- |
|  |  |  |  |  |  |  |
| chr18 | 35098249 | Hspa9 | HSPA9 | C>A | p.A651S | LUSC_T2 |
| chr18 | 35098249 | Hspa9 | HSPA9 | C>A | p.A651S | LUSC_T5 |
| chr18 | 35098249 | Hspa9 | HSPA9 | C>A | p.A651S | LUSC_T6 |
| chr18 | 35098249 | Hspa9 | HSPA9 | C>A | p.A651S | LUSC_T10 |
| chr18 | 35098249 | Hspa9 | HSPA9 | C>A | p.A651S | LUSC_T12 |
| chr18 | 35098249 | Hspa9 | HSPA9 | C>A | p.A651S | LUSC_T13 |
| chr18 | 35098249 | Hspa9 | HSPA9 | C>A | p.A651S | LUSC_T16 |
| chr10 | 68808901 | Cdk1 | CDK1 | C>A | p.S39I | LUSC_T4 |
| chr10 | 68808901 | Cdk1 | CDK1 | C>A | p.S39I | LUSC_T6 |
| chr10 | 68808901 | Cdk1 | CDK1 | C>A | p.S39I | LUSC_T7 |
| chr10 | 68808901 | Cdk1 | CDK1 | C>A | p.S39I | LUSC_T9 |
| chr10 | 68808901 | Cdk1 | CDK1 | C>A | p.S39I | LUSC_T13 |
| chr10 | 68808901 | Cdk1 | CDK1 | C>A | p.S39I | LUSC_T16 |
| chr18 | 37634751 | Pcdhb15 | PCDHB15 | C>T | p.R461C | LUSC_T2 |
| chr18 | 37634751 | Pcdhb15 | PCDHB15 | C>T | p.R461C | LUSC_T5 |
| chr18 | 37634751 | Pcdhb15 | PCDHB15 | C>T | p.R461C | LUSC_T10 |
| chr18 | 37634751 | Pcdhb15 | PCDHB15 | C>T | p.R461C | LUSC_T11 |
| chr18 | 37634751 | Pcdhb15 | PCDHB15 | C>T | p.R461C | LUSC_T14 |
| chr18 | 37634751 | Pcdhb15 | PCDHB15 | C>T | p.R461C | LUSC_T16 |
| chr13 | 60899872 | Ctsll3 | CTSL | G>A | p.P329S | LUSC_T2 |
| chr13 | 60899872 | Ctsll3 | CTSL | G>A | p.P329S | LUSC_T5 |
| chr13 | 60899872 | Ctsll3 | CTSL | G>A | p.P329S | LUSC_T6 |
| chr13 | 60899872 | Ctsll3 | CTSL | G>A | p.P329S | LUSC_T10 |
| chr13 | 60899872 | Ctsll3 | CTSL | G>A | p.P329S | LUSC_T16 |
| chr15 | 78720280 | Gga1 | GGA1 | G>A | p.D358N | LUSC_T3 |
| chr15 | 78720280 | Gga1 | GGA1 | G>A | p.D358N | LUSC_T5 |
| chr15 | 78720280 | Gga1 | GGA1 | G>A | p.D358N | LUSC_T10 |
| chr15 | 78720280 | Gga1 | GGA1 | G>A | p.D358N | LUSC_T11 |
| chr15 | 78720280 | Gga1 | GGA1 | G>A | p.D358N | LUSC_T12 |
| chr17 | 12915039 | Igf2r | IGF2R | T>C | p.N474D | LUSC_T4 |
| chr17 | 12876728 | Igf2r | IGF2R | C>A | p.V2434F | LUSC_T6 |
| chr17 | 12915039 | Igf2r | IGF2R | T>C | p.N474D | LUSC_T7 |
| chr17 | 12876728 | Igf2r | IGF2R | C>A | p.V2434F | LUSC_T11 |
| chr17 | 12876727 | Igf2r | IGF2R | A>G | p.V2434A | LUSC_T16 |
| chr15 | 78312103 | Il2rb | IL2RB | G>T | p.R475S | LUSC_T3 |
| chr15 | 78312103 | Il2rb | IL2RB | G>T | p.R475S | LUSC_T5 |
| chr15 | 78312103 | Il2rb | IL2RB | G>T | p.R475S | LUSC_T10 |
| chr15 | 78312103 | Il2rb | IL2RB | G>T | p.R475S | LUSC_T11 |
| chr15 | 78312103 | Il2rb | IL2RB | G>T | p.R475S | LUSC_T12 |
| chr17 | 71110690 | Dlgap1 | DLGAP1 | G>A | p.A329T | LUSC_T4 |
| chr17 | 71110690 | Dlgap1 | DLGAP1 | G>A | p.A329T | LUSC_T8 |
| chr17 | 71110690 | Dlgap1 | DLGAP1 | G>A | p.A329T | LUSC_T12 |
| chr17 | 71110690 | Dlgap1 | DLGAP1 | G>A | p.A329T | LUSC_T13 |
| chr10 | 32049645 | Nkain2 | NKAIN2 | C>G | p.V67L | LUSC_T6 |
| chr10 | 32049645 | Nkain2 | NKAIN2 | C>G | p.V67L | LUSC_T8 |
| chr10 | 32049645 | Nkain2 | NKAIN2 | C>G | p.V67L | LUSC_T9 |
| chr10 | 32049645 | Nkain2 | NKAIN2 | C>G | p.V67L | LUSC_T15 |
| chr11 | 101968542 | Pyy | PYY | G>A | p.P42L | LUSC_T1 |
| chr11 | 101968542 | Pyy | PYY | G>A | p.P42L | LUSC_T6 |
| chr11 | 101968542 | Pyy | PYY | G>A | p.P42L | LUSC_T8 |
| chr11 | 101968542 | Pyy | PYY | G>A | p.P42L | LUSC_T16 |
| chrX | 131293308 | Armcx3 | ARMCX3 | C>A | p.D59E | LUSC_T2 |
| chrX | 131293308 | Armcx3 | ARMCX3 | C>A | p.D59E | LUSC_T7 |
| chrX | 131293308 | Armcx3 | ARMCX3 | C>A | p.D59E | LUSC_T9 |
| chr1 | 132346000 | Cd55 | CD55 | C>T | p.V333I | LUSC_T7 |
| chr1 | 132346000 | Cd55 | CD55 | C>T | p.V333I | LUSC_T8 |
| chr1 | 132346000 | Cd55 | CD55 | C>T | p.V333I | LUSC_T10 |
| chr1 | 133914552 | Elk4 | ELK4 | C>T | p.P197L | LUSC_T4 |
| chr1 | 133914552 | Elk4 | ELK4 | C>T | p.P197L | LUSC_T7 |
| chr1 | 133914552 | Elk4 | ELK4 | C>T | p.P197L | LUSC_T10 |
| chr11 | 95887964 | Gip | GIP | G>A | p.R38Q | LUSC_T6 |
| chr11 | 95887964 | Gip | GIP | G>A | p.R38Q | LUSC_T8 |
| chr11 | 95887964 | Gip | GIP | G>A | p.R38Q | LUSC_T16 |
| chr4 | 119804939 | Hivep3 | HIVEP3 | T>C | p.L1994P | LUSC_T3 |
| chr4 | 119804939 | Hivep3 | HIVEP3 | T>C | p.L1994P | LUSC_T6 |
| chr4 | 119804939 | Hivep3 | HIVEP3 | T>C | p.L1994P | LUSC_T7 |
| chr8 | 25702060 | Ido1 | IDO1 | C>T | p.E26K | LUSC_T7 |
| chr8 | 25702060 | Ido1 | IDO1 | C>T | p.E26K | LUSC_T9 |
| chr8 | 25702060 | Ido1 | IDO1 | C>T | p.E26K | LUSC_T14 |
| chr9 | 75236064 | Mapk6 | MAPK6 | ->CCCCA | p.E653fs | LUSC_T1 |
| chr9 | 75236064 | Mapk6 | MAPK6 | ->CCCCA | p.E653fs | LUSC_T10 |
| chr9 | 75236064 | Mapk6 | MAPK6 | ->CCCCA | p.E653fs | LUSC_T11 |
| chr11 | 67017948 | Myh1 | MYH1 | A>C | p.T211P | LUSC_T2 |
| chr11 | 67017948 | Myh1 | MYH1 | A>C | p.T211P | LUSC_T4 |
| chr11 | 67017948 | Myh1 | MYH1 | A>C | p.T211P | LUSC_T5 |
| chr13 | 19715342 | Sfrp4 | SFRP4 | G>A | p.R14H | LUSC_T4 |
| chr13 | 19715342 | Sfrp4 | SFRP4 | G>A | p.R14H | LUSC_T5 |
| chr13 | 19715342 | Sfrp4 | SFRP4 | G>A | p.R14H | LUSC_T14 |
| chr17 | 47923040 | Tfeb | TFEB | ->CAG | p.Q94delinsQQ | LUSC_T5 |
| chr17 | 47923040 | Tfeb | TFEB | ->CAG | p.Q94delinsQQ | LUSC_T8 |
| chr17 | 47923040 | Tfeb | TFEB | ->CAG | p.Q94delinsQQ | LUSC_T10 |
| chr11 | 73073911 | Trpv1 | TRPV1 | A>T | p.T808S | LUSC_T3 |
| chr11 | 73073911 | Trpv1 | TRPV1 | A>T | p.T808S | LUSC_T9 |
| chr11 | 73073911 | Trpv1 | TRPV1 | A>T | p.T808S | LUSC_T15 |
| chr2 | 44844272 | Zeb2 | ZEB2 | C>A | p.D1157Y | LUSC_T1 |
| chr2 | 44844272 | Zeb2 | ZEB2 | C>A | p.D1157Y | LUSC_T5 |
| chr2 | 44844272 | Zeb2 | ZEB2 | C>A | p.D1157Y | LUSC_T10 |
| chr4 | 53063823 | Abca1 | ABCA1 | G>A | p.T1586M | LUSC_T9 |
| chr4 | 53063823 | Abca1 | ABCA1 | G>A | p.T1586M | LUSC_T14 |
| chr5 | 8947308 | Abcb4 | ABCB4 | G>A | p.R929Q | LUSC_T8 |
| chr5 | 8947308 | Abcb4 | ABCB4 | G>A | p.R929Q | LUSC_T16 |
| chr2 | 118457560 | Bub1b | BUB1B | T>A | p.L726I | LUSC_T1 |
| chr2 | 118457560 | Bub1b | BUB1B | T>A | p.L726I | LUSC_T7 |
| chr1 | 34239291 | Dst | DST | G>A | p.A2444T | LUSC_T4 |
| chr1 | 34239291 | Dst | DST | G>A | p.A2444T | LUSC_T6 |
| chr8 | 125829746 | Fanca | FANCA | C>T | p.G300R | LUSC_T8 |
| chr8 | 125829746 | Fanca | FANCA | C>T | p.G300R | LUSC_T16 |
| chr5 | 148511484 | Flt1 | FLT1 | A>G | p.F89L | LUSC_T4 |
| chr5 | 148511484 | Flt1 | FLT1 | A>G | p.F89L | LUSC_T13 |
| chr8 | 74209321 | Jak3 | JAK3 | C>T | p.P839L | LUSC_T8 |
| chr8 | 74209321 | Jak3 | JAK3 | C>T | p.P839L | LUSC_T10 |
| chr6 | 71561948 | Kdm3a | KDM3A | G>A | p.P357L | LUSC_T2 |
| chr6 | 71561948 | Kdm3a | KDM3A | G>A | p.P357L | LUSC_T11 |
| chr2 | 165880571 | Ncoa3 | NCOA3 | G>A | p.S594N | LUSC_T1 |
| chr2 | 165880571 | Ncoa3 | NCOA3 | G>A | p.S594N | LUSC_T3 |
| chr1 | 152302105 | Prg4 | PRG4 | C>G | p.R649P | LUSC_T8 |
| chr1 | 152302052 | Prg4 | PRG4 | C>T | p.E667K | LUSC_T16 |
| chr17 | 44872394 | Runx2 | RUNX2 | ->CTG | p.E64delinsQE | LUSC_T9 |
| chr17 | 44872394 | Runx2 | RUNX2 | ->CTG | p.E64delinsQE | LUSC_T16 |
| chr3 | 93455535 | Tdpoz2 | SPOP | ->CT | p.C351fs | LUSC_T6 |
| chr3 | 93455535 | Tdpoz2 | SPOP | ->CT | p.C351fs | LUSC_T16 |
| chr2 | 165638202 | Zmynd8 | ZMYND8 | C>T | p.R646Q | LUSC_T1 |
| chr2 | 165638202 | Zmynd8 | ZMYND8 | C>T | p.R646Q | LUSC_T3 |
| chr8 | 86265030 | Adgre5 | ADGRE5 | C>T | p.G3S | LUSC_T16 |
| chr1 | 38266735 | Aff3 | AFF3 | T>C | p.N712S | LUSC_T4 |
| chr18 | 34455734 | Apc | APC | G>T | p.S293I | LUSC_T4 |
| chr9 | 95810937 | Atr | ATR | G>A | p.R1631H | LUSC_T4 |
| chr7 | 20394577 | Bcl3 | BCL3 | C>T | p.R359Q | LUSC_T9 |
| chr10 | 21868439 | E030030I06Rik | CDK6 | ->A | p.A127fs | LUSC_T7 |
| chr5 | 137041260 | Cux1 | CUX1 | ->CTCC | p.A103fs | LUSC_T7 |
| chr15 | 82447637 | Cyp2d34 | CYP2D6 | T>G | p.T307P | LUSC_T10 |
| chr18 | 71495728 | Dcc | DCC | G>A | p.P1086L | LUSC_T10 |
| chr7 | 135115721 | Fus | FUS | C>A | p.S143R | LUSC_T13 |
| chr11 | 74982955 | Hic1 | HIC1 | A>G | p.S23P | LUSC_T3 |
| chr1 | 138365574 | Kif14 | KIF14 | G>A | p.R347Q | LUSC_T4 |
| chr15 | 98680018 | Kmt2d | KMT2D | ->TGC | p.H3285delinsQH | LUSC_T5 |
| chr9 | 109973541 | Map4 | MAP4 | ->A | p.E930fs | LUSC_T4 |
| chr11 | 61303714 | Mapk7 | MAPK7 | ->GCTGGT | p.A590delinsAPA | LUSC_T1 |
| chr17 | 84188368 | Mta3 | MTA3 | G>T | p.C378F | LUSC_T5 |
| chr13 | 3883615 | Net1 | NET1 | G>A | p.R443C | LUSC_T8 |
| chr15 | 8246761 | Nipbl | NIPBL | C>T | p.G2455S | LUSC_T15 |
| chr11 | 58889698 | Obscn | OBSCN | T>A | p.D2868V | LUSC_T3 |
| chr2 | 153058120 | Plagl2 | PLAGL2 | C>T | p.R199Q | LUSC_T2 |
| chr2 | 29557961 | Rapgef1 | RAPGEF1 | T>C | p.S444P | LUSC_T4 |
| chr9 | 96378958 | Rnf7 | RNF7 | G>A | p.S15L | LUSC_T5 |
| chr1 | 85253984 | C130026I21Rik | SP140 | C>T | p.R143K | LUSC_T14 |
| chr4 | 114714032 | Stil | STIL | G>A | p.V859I | LUSC_T16 |
| chr12 | 106440714 | Tcl1b4 | TCL1B | G>A | p.C9Y | LUSC_T8 |
| chr11 | 60960519 | Tnfrsf13b | TNFRSF13B | C>A | p.R175S | LUSC_T10 |
